# Unlocking the Anti-Breast Cancer Potential of *Aralia chinensis* L.

**DOI:** 10.3390/cimb47080662

**Published:** 2025-08-16

**Authors:** Juan Xue, Lei Li, Yongjia Shu, Chengshi Xie, Tian Lu, Huifang Chai

**Affiliations:** School of Pharmacy, Guizhou University of Traditional Chinese Medicine, Guiyang 550025, China; xuejuan062@gzy.edu.cn (J.X.); leiflee1998@sina.com (L.L.); 18083163187@163.com (Y.S.); xie177856@163.com (C.X.); kkllttyy2025@163.com (T.L.)

**Keywords:** *Aralia chinensis* L., triterpenoid saponins, UPLC-Q Exactive Orbitrap-MS, serum pharmacochemistry, breast cancer

## Abstract

*Aralia chinensis* L. has shown potential in breast cancer treatment, yet its pharmacodynamically active components and mechanisms remain undefined. To systematically identify the bioactive constituents absorbed into the bloodstream and elucidate their multi-target mechanisms against breast cancer, we employed ultra-high-performance liquid chromatography in conjunction with Q Exactive Orbitrap mass spectrometry (UHPLC-Q Exactive Orbitrap-MS) alongside serum pharmacochemistry to analyze the chemical constituents of total saponins derived from *A. chinensis* (TSAC) and to identify the blood-absorbed prototypes in a rat model. Network pharmacology predicted targets and pathways of serum prototypes, validated by molecular docking and in vitro experiments. We identified 38 triterpenoid saponins, 3 steroidal saponins, and 8 triterpenoids in TSAC, with 22 prototype compounds detected in serum. An integrative analysis encompassing 486 compound targets and 1747 genes associated with breast cancer elucidated critical pathways, notably the PI3K-Akt signaling pathway and resistance mechanisms to EGFR tyrosine kinase inhibitors. Molecular docking confirmed strong binding of araloside A and elatoside L to *SRC*, *PIK3R1*, *PIK3CA*, *STAT3*, and *EGFR*. In MCF-7 cells, TSAC suppressed proliferation and migration while downregulating *Src*, *PI3K*, and *EGFR* expression at the gene and protein levels. This study successfully identified TSAC’s serum-absorbed bioactive components and demonstrated their anti-breast cancer effects via multi-target mechanisms involving the *Src/PI3K/EGFR* axis, providing a crucial pharmacological foundation for developing *A. chinensis*-derived breast cancer therapies.

## 1. Introduction

Breast cancer has overtaken lung cancer as the most frequently diagnosed malignancy and is now the second leading cause of cancer-related mortality among women worldwide [1]. In 2022, there were approximately 340,000 new cases and 43,250 deaths attributed to this disease [2]. In China, both the incidence and mortality rates are escalating and are projected to increase by 36.27% and 54.01%, respectively [3]. While current therapies (endocrine therapy, targeted agents, etc.) have improved patient outcomes [4,5,6,7], critical challenges persist: the rapid development of drug resistance (e.g., ER signaling reactivation via ERα mutations or PI3K/AKT pathway activation in tamoxifen resistance [8,9], acquired resistance to *HER2*-targeted therapies [8]), tumor spatiotemporal heterogeneity (molecular subtype variations [9], clonal evolutionary diversity [10]), and treatment-related toxicities (anthracycline-induced cardiotoxicity [11], immune checkpoint inhibitor-related autoimmunity [12]). To address these limitations, multi-target integrative strategies demonstrate therapeutic potential: bioactive phytocompounds, such as ginsenosides, baicalein, and curcumin, exert coordinated anti-tumor effects through proliferation inhibition (G1/S phase arrest), apoptosis induction (Caspase-3 activation), chemoresistance reversal (P-gp efflux pump suppression), and metastasis blockade (*EMT/VEGF* modulation) [13,14,15]. These effects are synergized with immune microenvironment remodeling (enhanced CD8+ T-cell infiltration/Treg suppression) and PI3K/Akt/mTOR pathway co-regulation [16,17], establishing dual tumoricidal–immunomodulatory mechanisms. Concurrently, their antioxidative stress capacity (*Nrf2* activation) and organ-protective properties mitigate treatment toxicity [18], achieving systematic “efficacy enhancement-toxicity reduction”.

### Botanical Description of Chinensis

*Aralia* species, abundant in antitumor triterpenoid saponins and terpenoids, represent a promising botanical candidate within this framework. Widely distributed in China, Japan, South Korea, and North America [19], *Aralia chinensis* L. is primarily found in Guizhou, Yunnan, Chongqing, and Sichuan provinces. Its roots, stems, and bark have been used medicinally for millennia, with buds consumed as wild vegetables in East Asia [20]. First documented in Sun Simiao’s Qian Jin Fang (Tang Dynasty, 652 CE) and detailed in the Compendium of Materia Medica (Ming Dynasty, 1578), this plant has historically been used to treat rheumatism, lumbosacral pain, and traumatic injuries. Modern studies identify triterpenoid saponins and terpenoids as its predominant bioactive phytochemicals [21], demonstrating anti-inflammatory, analgesic, antitumor, hepatoprotective, and metabolic regulatory activities, as well as cardiovascular and neuroprotective effects [22]. Recent investigations highlight their therapeutic potential against breast cancer [23,24,25,26,27,28].

In the current investigation, UPLC-Q Exactive Orbitrap-MS was employed to identify the chemical constituents of the total saponins derived from *A. chinensis* (TSAC) in vitro, as well as the prototype components that are absorbed into rat serum. By synthesizing serum pharmacochemistry with network pharmacology, this study seeks to elucidate the potential targets and mechanisms through which TSAC may exert therapeutic effects in the treatment of breast cancer, with particular emphasis on the prototype constituents that enter the circulatory system. Molecular docking validated the predicted interactions, and in vitro experiments further confirmed the analytical results. This research seeks to decipher the in vivo action mechanism of TSAC and its therapeutic efficacy in breast cancer, providing a scientific basis for its clinical application and natural product-derived drug development.

## 2. Materials and Methods

### 2.1. Materials and Reagents

Samples of *Aralia* were collected from Jianhe County, Guizhou Province, China, and authenticated as the root bark of *Aralia chinensis* L. by Professor Qingwen Sun (Medicinal Plant Taxonomist, Guizhou University of Traditional Chinese Medicine) through diagnostic morphological characteristics including asymmetrically based leaflets in 2–3-pinnately compound leaves, densely tawny-tomentose branches of paniculate inflorescences, yellowish-white pentamerous flowers, and globose 5-ridged berries, with supplementary stereomicroscopic verification of leaf epidermal trichome bases. Taxonomic identification rigorously referenced Flora Reipublicae Popularis Sinicae [29] and Iconographia Cormophytorum Sinicorum [30], while current nomenclature was confirmed via Plants of the World Online (POWO ID: urn:lsid:ipni.org:names:89710-1) and IPNI (ID: 89710-1) in January 2023. Authenticated samples were stored at 4 °C in our laboratory. Reagents included: HPLC-grade methanol, acetonitrile, and formic acid (Thermo Fisher, Shanghai, China); ultrapure water (Watson, Guangzhou, China); fetal bovine serum (Fuheng Biotechnology, Shanghai, China); Minimum Essential Medium (Thermo Fisher Scientific, Shanghai, China); CCK-8 assay kit (MedChemExpress, Shanghai, China); and FastStart Universal SYBR Green Master Mix (Rox; Roche, Switzerland).

### 2.2. Preparation of TSAC

A total of 5 kg of powdered root bark samples from *A. chinensis* were suspended in ten volumes of 70% ethanol and subjected to reflux extraction three times, with each extraction lasting 2 h. The selection of 2 h as the duration for each extraction was based on the results of preliminary pre-experiments: when the extraction time was less than 2 h, the extraction rate of the target active components increased significantly with the extension of time; however, after reaching 2 h, prolonging the extraction time resulted in no statistically significant difference in the extraction rate of the components (*p* > 0.05). Moreover, excessive prolongation of the extraction time would increase energy consumption and the risk of impurity dissolution. Therefore, 2 h is the optimal choice that balances extraction efficiency, component integrity, and experimental economy. Subsequently, the resulting extract was filtered and concentrated at a temperature of 45 °C under reduced pressure to achieve a suitable volume. This concentrated extract was then diluted with deionized water to create an aqueous solution with a concentration of 1 g/mL, corresponding to the original quantity of the medicinal material. An AB-8 macroporous resin (3 kg) was used to purify the aqueous extract, and 20%, 50%, 70%, and 95% alcohol were used for elution. 50% and 70% eluents were collected and condensed in an alcohol-free solution. The aqueous eluent underwent lyophilization to yield a total saponins powder, which was subsequently stored at −20 °C for future applications. The freeze-drying process was conducted as follows: initially, the aqueous eluent was pre-frozen at −80 °C for 12 h. The primary drying stage followed this in a freeze-drying apparatus under vacuum at −30 °C. The duration of primary drying (48 to 72 h) was determined based on the visual observation of complete ice sublimation from the samples and stabilization of the chamber pressure, indicating the endpoint of this critical phase. Subsequently, the secondary drying stage was performed at temperatures ranging from 20 to 25 °C for 24 h to remove residual bound water. The average content of total saponins was determined to be 76.7%, as measured using a UV-5500H UV-Vis spectrophotometer (Metash, Shanghai, China), by previously established methodologies [31].

### 2.3. Preparation of Samples for LC-MS/MS Analysis

A precise quantity of 40.5 mg of TSAC was measured and placed into a 1.5 mL Eppendorf tube, to which 400 μL of Wahaha purified water was added in order to create a solution with a concentration of 100 mg/mL of the crude drug. The resulting mixture was subjected to vortexing for 2 min, followed by extraction through ultrasonication for 30 min. Subsequently, the tube containing the aqueous sample was centrifuged at 13,000 rpm for 30 min at a temperature of 4 °C. The supernatant obtained was then filtered using a 0.22 μm aqueous phase filter membrane in preparation for LC-MS/MS analysis.

### 2.4. Animals

Twenty-four male Sprague Dawley rats (6–8 weeks old, 220 ± 20 g, SPF grade) were acquired from Changsha Tianqin Biotechnology Co., Ltd.,a Chinese company (CHN). under the animal license SCXK (Xiang) 2022-0011. The rats were maintained in an environment with a temperature of 24 ± 2 °C and a humidity level of 60–70%. They were provided with ad libitum access to food and water and were allowed a one-week acclimatization period to a 12:12 h light-dark cycle. All experimental protocols received approval from the Animal Ethics Committee of Guizhou University of Traditional Chinese Medicine (SYXK (Qian) 2021-0005). Prior to the commencement of the experiments, the rats underwent a 12 h fasting period while having unrestricted access to water. Subsequently, the rats were randomly assigned to either control or treatment groups.

### 2.5. Preparation of Medicated Serum Samples

Serum samples were obtained from 24 rats that were administered TSAC (3.6 g/kg body weight, diluted in a 0.5% carboxymethyl cellulose aqueous solution) at predetermined intervals. Control serum samples were collected from six rats that received an equivalent volume of the 0.5% sodium carboxymethyl cellulose solution. The treatment was administered bi-daily over four consecutive days. Blood samples, approximately 1 mL each, were drawn from the oculi chorioideae vein at intervals of 0.5, 1, 1.5, 2, 3, 4, 5, and 6 h following the final TSAC administration. The collected blood samples were subjected to centrifugation at 3000 rpm for 15 min at 4 °C. The supernatants from the TSAC group samples were pooled and stored at −80 °C. For protein precipitation, 150 μL of serum was combined with 450 μL of a methanol–acetonitrile mixture (*v*:*v* = 2:1), which contained L-2-chlorophenylalanine at a concentration of 2 μg/mL. The precipitation protocol involved vortexing the sample for 1 min, followed by ultrasonic extraction in an ice-water bath for 10 min, and then maintaining the sample at −40 °C for 30 min. After centrifugation at 12,000 rpm for 10 min at 4 °C, the sample was kept at −40 °C for an additional 120 min. Subsequently, 500 μL of the supernatant was transferred to a new tube and evaporated to dryness. The resulting residue was reconstituted in 125 μL of a water–methanol–acetonitrile solution (*v*:*v:v* = 1:2:1), vortexed for 1 min, subjected to ultrasonic extraction for 3 min, and then stored overnight at −40 °C. Following this, the residue was centrifuged at 12,000 rpm for 10 min at 4 °C. Finally, 100 μL of the supernatant was placed into an LC-MS vial equipped with a pin-lined tube for subsequent analysis. Quality control (QC) samples were generated by combining equal volumes of the supernatants from the control, treatment, and TSAC groups.

### 2.6. UPLC-Q-Exactive Orbitrap MS Condition

Chemical analyses were conducted utilizing an ACQUITY UPLC I-Class plus UPLC system (Waters, Milford, MA, USA) in conjunction with a Q-Exactive Orbitrap high-resolution mass spectrometer (Thermo, Waltham, MA, USA). Sample separation was achieved on an ACQUITY UPLC HSS T3 column (100 mm × 2.1 mm, 1.8 μm) at a flow rate of 0.35 mL/min, with an injection volume of 5 μL. The column was maintained at a temperature of 45 °C. A gradient elution program was implemented using a 0.1% formic acid solution in water (designated as phase A) and acetonitrile (designated as phase B). The gradient elution protocol was as follows: from 0 to 2 min, 5% B; from 2 to 4 min, a transition from 5% to 30% B; from 4 to 8 min, a transition from 30% to 50% B; from 8 to 10 min, a transition from 50% to 80% B; from 10 to 14 min, a transition from 80% to 100% B; from 14 to 15 min, 100% B; from 15 to 15.1 min, a transition from 100% to 5% B; and from 15.1 to 16 min, maintaining at 5% B.

Mass spectrometry was conducted utilizing a Q Exactive Orbitrap mass spectrometer, which was outfitted with a H-ESI. The voltages for positive and negative ion modes were established at 3800 V and 3000 V, respectively. The capillary temperature was maintained at 320 °C, while the auxiliary gas heater temperature was set to 350 °C. The flow rate of the sheath gas was calibrated to 35 arbitrary units (Arb), and the auxiliary gas flow rate was adjusted to 8 Arb. The radiofrequency (RF) level of the S-lens was configured to 50. The full mass spectrometry scan encompassed a mass-to-charge ratio (*m*/*z*) range of 100 to 1500, with the Orbitrap mass spectrometer achieving a resolution of 70,000. Tandem mass spectrometry (MS/MS) was performed in a data-dependent ms2 mode at a resolution of 17,500, with the collision energy applied in step mode at 10, 20, and 40 eV.

### 2.7. Data Processing and Chemical Identification

Progenesis QI V2.3 (Nonlinear Dynamics, Newcastle, UK) was employed to analyze the original Thermo RAW data, facilitating processes such as baseline filtering, peak identification, integration, retention time correction, peak alignment, and normalization. The tolerance thresholds for precursor and product ions were established at five ppm and 10 ppm, respectively. Compound identification was matched to the LuMet-TCM database, a self-built database established by Luming Biological Technology Co., Ltd. (Shanghai, China), which consists of the retention time, precise mass ions, and MS2 fragments of >5000 standard references, including alkaloids, phenolic acids, flavonoids, coumarins, phenylpropanoids, lignans, terpenoids, and saponins. The MS2 spectral data compared with standards from the LuMet-TCM database are mainly developed on three dimensions: a, the error of retention time is within ±0.2 min; b, the error of precursor ions is within five ppm; c, the fragment ions of measured compounds and standards were compared. Based on comparing the chemical name, molecular formula, exact mass, chemical structure, and possible fragmentation patterns with those reported in the literature, the chemical constituents documented no data matrices for standards in the LuMet-TCM database.

### 2.8. Network Analysis

#### 2.8.1. Collection of Targets for Active Ingredients

The compounds detected in the serum samples of rats exhibit significant potential for the treatment of a variety of medical conditions. The structural representations of the prototype compounds were obtained from the PubChem database (https://pubchem.ncbi.nlm.nih.gov/, accessed on 16 June 2023) or generated utilizing ChemDraw software (version 18.0). The SwissTargetPrediction database (http://www.swisstargetprediction.ch) was employed to identify their biological targets by utilizing SMILES notation and specifying ‘Homo sapiens’ as the organism.

#### 2.8.2. Collection of Breast Cancer-Related Genes

Genes associated with breast cancer were collected from the Comparative Toxicogenomics Database (CTD) (http://ctdbase.org/), Disease Gene Network (DisGeNET) (http://www.disgenet.org/, accessed on 20 June 2023), GeneCards (https://www.genecards.org/, accessed on 21 June 2023), and Online Mendelian Inheritance in Man (OMIM) databases. Based on the frequency of genes in the four databases, genes that appeared two or more times were considered candidate genes for breast cancer.

#### 2.8.3. Construction of Active Compound–Target and Protein–Protein Interaction (PPI) Networks

Active ingredients and their targets were imported into Cytoscape (version 3.7.2) to construct an interaction network. The overlapping targets of the bioactive compounds that were absorbed, along with breast cancer, were analyzed using the STRING online platform (https://cn.string-db.org/, accessed on 24 June 2023) for protein–protein interaction (PPI) analysis. The organism was specified as ‘Homo sapiens,’ and a confidence score threshold of greater than 0.9 was applied. Single nodes were excluded from the analysis, while all other parameters were maintained at their default settings to generate the protein interaction network. The results were imported into Cytoscape (version 3.7.2) for network analysis. The key genes of the top 100 and top 20 targets calculated by degree values were extracted using the CytoHubba plug-in for visual network analysis.

#### 2.8.4. Analyses of the GO Functional Enrichment and KEGG Pathway

Gene Ontology (GO) enrichment analysis and Kyoto Encyclopedia of Genes and Genomes (KEGG) pathway analysis was conducted utilizing the DAVID database, focusing on the targets of active ingredients and overlapping targets associated with absorbed bioactive compounds in breast cancer (https://david.ncifcrf.gov/, accessed on 1 July 2023) through the “functional annotation” module. The identifiers and species selected for this analysis were “OFFICIAL_GENE_SYMBOL” and Homo sapiens, respectively. Results were filtered for statistical significance at *p* < 0.05, and the top 20 findings from both the GO enrichment and KEGG pathway analyses were visualized using OECloud tools (https://cloud.oebiotech.com).

#### 2.8.5. Molecular Docking

The chemical structures of 22 absorbed active ingredients were selected as ligands, drawn with ChemDraw 18.0, and saved in SDF format. Additionally, *SRC* (PDBID: 6ATE), *PIK3R1* (PDBID: 1H90), *PIK3CA* (PDBID: 2RDO), *STAT3* (PDBID: 6NJS), and *EGFR* (PDBID: 1XKK) were selected as receptor proteins and downloaded from the Protein Data Bank database (https://www.rcsb.org/, accessed on 10 July 2023). The Protein Preparation Wizard module in Schrödinger was used to add hydrogen atoms, assign H-bonds, remove water molecule ligands, and minimize energy in the proteins. The LigPrep module was used to obtain the 3D structures of the compounds and perform conformation optimization and energy minimization. The Receptor Grid Generation module was employed to create docking grids. Subsequently, molecular docking of the compounds and proteins was conducted utilizing the ligand-docking module. A binding affinity value of less than −5 kcal/mol indicated a significant binding interaction between the proteins and ligands. Finally, the docking results were analyzed and visualized using PyMOL software (version 2.4.0).

### 2.9. Experimental Validation In Vitro

#### 2.9.1. Cell Culture

The Michigan Cancer Foundation-7 (MCF-7; Catalog Number FH0215) cell line was acquired from Shanghai Fuheng Biotechnology Co., Ltd., located in Shanghai, China. The MCF-7 cells were cultured in MEM supplemented with 10% FBS and 1% penicillin/streptomycin and were incubated at 37 °C in a humidified atmosphere containing 5% carbon dioxide.

#### 2.9.2. Cell Viability Assay

In the present study, breast cancer cells in the exponential growth phase were utilized for all experimental procedures. MCF-7 cell suspensions were plated in 96-well plates at a density of 5 × 10^3^ cells per well and incubated overnight. Subsequently, the cells were exposed to varying concentrations of TSAC (0, 0.1, 0.2, 0.3, 0.4, and 0.5 mg/mL), with three replicates for each concentration, for durations of 24 h and 48 h to capture time-dependent cytotoxic effects and assess both acute (24 h) and sustained (48 h) responses, consistent with standard protocols for evaluating anti-proliferative agents. Following treatment, 100 μL of MEM supplemented with 10% CCK-8 solution was added to each well and incubated for one hour. The impact of TSAC on cell viability was assessed by measuring the optical density at 450 nm using a microplate reader (Model: LD-96A; Manufacturer: Shandong Laende, Lanling, China.).

#### 2.9.3. Colony Formation Assay

MCF-7 cells were cultured in 6-well plates at a density of 700 cells per well and allowed to incubate for 24 h. Following this incubation period, the cells were exposed to varying concentrations of TSAC (0, 0.1, 0.2, 0.3, and 0.4 mg/mL) for an additional 24 h. After treatment, the medium containing TSAC was discarded, and the cells were washed with PBS before being maintained in a complete medium for a subsequent period of seven days. After this incubation, the cells that had proliferated were fixed using a 4% paraformaldehyde solution and subsequently stained with 0.5% crystal violet at room temperature for 30 min. Following a rinse with PBS, the number of colonies formed was quantified.

#### 2.9.4. Migration Assay

MCF-7 cells were cultured in 6-well plates at a density of 5 × 10^5^ cells per well and allowed to incubate for 24 h. A micropipette tip was employed to create a wound gap in the cell monolayer, after which the wells were rinsed with PBS to eliminate any detached cells. Subsequently, the cells were exposed to serum-free medium containing TSAC at concentrations of 0, 0.1, 0.2, and 0.3 mg/mL for 24 h in a humidified incubator maintained at 37 °C with 5% CO2. Concurrently, photographic documentation was conducted under a microscope at 0, 12, and 24 h.

### 2.10. Real-Time Quantitative Polymerase Chain Reaction

MCF-7 cells were cultured in 6-well plates at a density of 5 × 10^5^ cells per well and allowed to incubate overnight. Subsequently, the cells were subjected to treatment with concentrations of 0, 0.3, and 0.4 mg/mL for a duration of 24 h. Total RNA was extracted utilizing TRIzol (Servicebio, Wuhan, China) in conjunction with chloroform (Sinopharm, Shanghai, China). The concentration of the extracted total RNA was determined through ultramicroscopic spectrophotometry using a NanoDrop 2000 (Thermo, Waltham, MA, USA). Real-time quantitative polymerase chain reaction (RT-qPCR) was conducted employing a RevertAid First Strand cDNA Synthesis Kit (Thermo Scientific, Waltham, MA, USA) and a SLAN real-time PCR system (Hongshitech, Shanghai, China). All primers utilized in the study were designed by Tsingke Biotech (Beijing, China). The relative expression levels of the genes were calculated using the 2^−ΔΔCt^ method, and the sequences of the primers employed in the RT-qPCR analysis are detailed in Table 1.

### 2.11. Western Blot Analysis

MCF-7 cells were cultured in 6-well plates at a density of 5 × 10^5^ cells per well and allowed to incubate overnight. Subsequently, the cells were treated with TSAC at concentrations of 0, 0.3, and 0.4 mg/mL for an additional 24 h. Following treatment, cell lysates were prepared using radioimmunoprecipitation assay buffer supplemented with protease and phosphatase inhibitors. The protein concentration of the lysates was determined using a bicinchoninic acid protein assay kit (Thermo, MA, USA) by the manufacturer’s instructions. Subsequently, the proteins were isolated through 10% sodium dodecyl sulfate-polyacrylamide gel electrophoresis and subsequently transferred to polyvinylidene fluoride membranes (Merck, Darmstadt, Germany). These membranes were then subjected to a blocking step with 5% skimmed milk at room temperature for one hour, followed by overnight incubation at 4 °C with primary antibodies specific to *Src* (1:5000, 60315-1-Ig, Proteintech, Wuhan, China), *PI3K* (1:5000, 82796-4-RR, Proteintech, Wuhan, China), *EGFR* (1:5000, 66455-1-Ig, Proteintech, Wuhan, China), and β-Actin (1:10,000, ab8226, Abcam, Cambridge, England). Following this, the membranes were incubated at room temperature for one hour with horseradish peroxidase-conjugated goat anti-rabbit IgG (1:3000, E-AB-1003; Elabscience, Wuhan, China) or horseradish peroxidase-conjugated goat anti-mouse IgG secondary antibody (1:3000, E-AB-1001; Elabscience). The immunoreactivities of the proteins were subsequently detected using an enhanced chemiluminescence (ECL) kit. Visualization of the blots was accomplished using a digital gel imaging system (ChemiDoc; Bio-Rad, Hercules, CA, USA), and the quantification of protein bands was performed utilizing ImageJ software (version 1.43).

### 2.12. Statistical Analysis

The data are expressed as mean ± standard deviation (SD). Comparisons between groups were conducted using either an unpaired Student’s *t*-test for two groups or one-way ANOVA followed by Tukey’s post hoc analysis, utilizing GraphPad Prism version 8.3.0 (GraphPad, San Diego, CA, USA). A *p*-value of less than 0.05 was deemed statistically significant, while “ns” denotes no significant difference. All experiments were performed a minimum of three times.

## 3. Results

### 3.1. Identification and Characterization of Chemical Compounds

**Figure 1:**
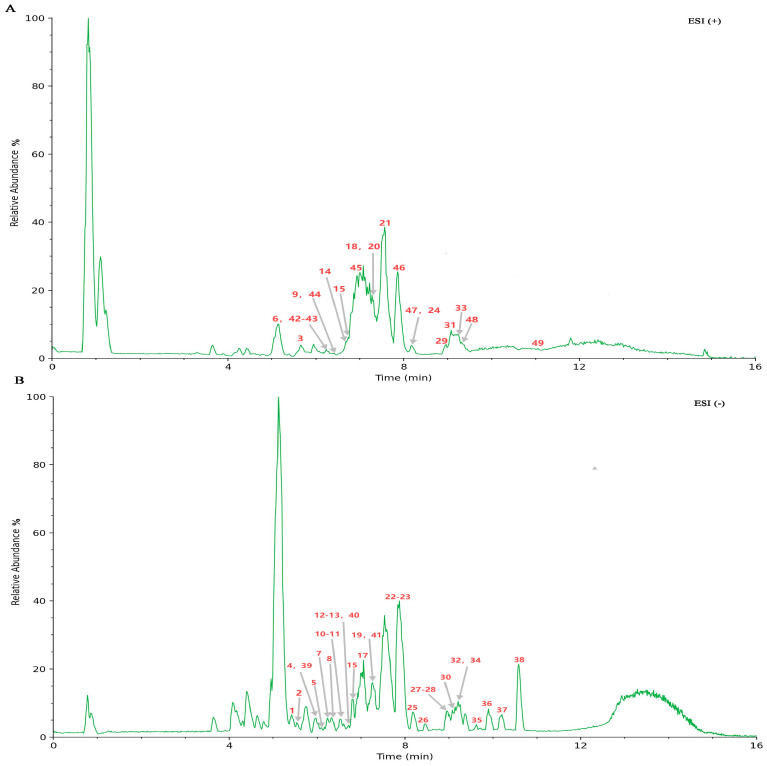
Base peak intensity (BPI) chromatograms of TSAC obtained using UPLC-Q Exactive-Orbitrap-MS analysis. Positive scan (**A**); Negative scan (**B**).

The content of TSAC was analyzed utilizing ultra-performance liquid chromatography coupled with UPLC-Q Exactive Orbitrap-MS. The BPI chromatograms for TSAC in both positive and negative ion modes are presented in Figure 1. Based on the abovementioned identification strategy, 49 chemical compounds were identified or putatively characterized in TSAC, including 38 triterpenoid saponins, three steroidal saponins, and eight triterpenoids (Table S1). In addition, 61 compounds with minor percentages, including five terpenes, 20 phenylpropanoids, five phenols, three alkaloids, five saccharides, 12 carboxylic and organic acids, and 11 amino acids and their derivatives, were identified, as listed in Table S2. The identification of several specific compounds is described below.

### 3.2. Triterpenoid Saponins

The aglycones of *Aralia* triterpenoid saponins consist of pentacyclic triterpenes. The presence of hydroxyl groups (-OH) facilitates the generation of fragmentation data for all aglycone forms, resulting in the formation of [aglycone-H_2_O+H]^+^ and [aglycone-2H_2_O+H]^+^ ions in the positive ionization mode, and conversely, in the negative ionization mode. The formation sites of the sugar chains of *Aralia* triterpenoid saponins are predominantly the C-3 and C-28 bonds, and the monosaccharide composition includes glucose, galactose, rhamnose, arabinose, xylose, and glucuronic acid. The neutral loss fragments were 162 Da (Glc/Gal), 146 Da (Rha), 132 Da (Ara/Xyl), and 176 Da (GlcA). In addition, complex glycosidic linkages composed of more than two monosaccharides included Hex + Hex (324 Da), Hex + HexA (338 Da), Hex + Pen + HexA (470 Da), and Hex + MePen (308 Da).

*Aralia* triterpenoid saponins are among the most valuable ingredients responsible for their pharmacological activities. The predominant dissociation mechanism observed in triterpenoid saponins involves the sequential elimination of glycosidic units at the C-3 and C-28 positions of aralosides, ultimately leading to the generation of [aglycone + H]^+^ or [aglycone − H]^−^ ions in either positive or negative ionization modes. The compound designated as H21, characterized by a pentacyclic carbon framework, was identified as araloside A.

The MS fragmentation rule for araloside A was comprehensively elucidated to provide a fundamental principle for facilitating the identification and characterization of *aralosides*, as shown in Figure S1A. Araloside A exhibited an [M+NH_4_]^+^ ion at *m*/*z* 944.5149 and an [aglycone-H_2_O+H]^+^ ion at *m*/*z* 439.3562 in positive ionization mode. Conversely, in negative ionization mode, araloside A presented a deprotonated ion [M-H]^−^ at *m*/*z* 925.4785, along with significant fragment ions at *m*/*z* 793.4370 [M-H-Ara]^−^, 763.4265 [M-H-Glc]^−^, 631.3853 [M-H-Glc-Ara]^−^, and 455.3527 [M-H-Glc-Ara-GlcA]^−^, which were generated as a result of MS2 collision energy. Compound H_2_O displayed a deprotonated ion [M-H]^−^ at *m*/*z* 955.4882, which was identified as ginsenoside Ro through comparison with a reference standard. The MS2 spectrum revealed characteristic peaks at *m*/*z* 793.4314 [M-H-Glc]^−^, 631.3846 [M-H-2Glc]^−^, and 455.3545 [M-H-2Glc-GlcA]^−^. Compound H26 demonstrated an excimer ion of [M-H]^−^ at *m*/*z* 941.5082 and yielded fragment ions at *m*/*z* 779.4579 [M-H-Glc]^−^, 617.4041 [M-H-2Glc]^−^, and 455.3535 [M-H-3Glc]^−^. This compound was tentatively identified as silphioside E.

### 3.3. Triterpenoids

Eight triterpenoids were identified in TSAC, including oleanolic acid sapogenin, hederagenin sapogenin, and caulophyllogenin sapogenin, all of which have a pentacyclic carbon skeleton. The main fragmentation rule of these components is the continuous loss of neutral units such as H_2_O, HCOOH, and CO_2_. Compound H49 was identified as hederagenin by comparing its fragmentation behavior with the standard’s. The proposed hederagenin fragmentation pathway is shown in Figure S1B. Compound H49 at *m*/*z* 473.3616 is attributed to the deprotonated ion [M+H]^+^, which formed fragment ions at *m*/*z* 455.3508 [M+H-H_2_O]^+^ and 437.3405 [M+H-2H_2_O]^+^ through the successive processes of dehydration. Compounds H48, H42, and H45, which exhibited fragmentation behavior similar to hederagenin, were identified as arjunolic acid, caulophyllogenin, and oleanolic acid, respectively. Compound H47 displayed an adduct ion [M+H-H_2_O]^+^ at *m*/*z* 455.3519 and formed characteristic peaks at *m*/*z* 437.3409 [M+H-2H_2_O]^+^ and 409.3452 [M+H-H_2_O-HCOOH]^+^. Based on the reported literature, they were putatively identified as echinocystic acid, an isomer of hederagenin [31]. The distinguishing strategy was based on the intensity ratio of *m*/*z* 437.3409 [M+H-2H_2_O]^+^ to *m*/*z* 455.3519 [M+H-H_2_O]^+^ in the ESI+-MS spectra. Hederagenin had a ratio of >2, whereas echinocystic acid had a ratio of <0.8.

### 3.4. Other Compounds

Other minor compounds in TSAC, including five terpenes, 20 phenylpropanoids, five phenols, three alkaloids, five saccharides, 12 carboxylic and organic acids, and 11 amino acids and their derivatives, were unintentionally identified using high-resolution UPLC-Q-Exactive-Orbitrap MS. Compound H67 was unambiguously identified as isochlorogenic acid C, a phenylpropanoid, through comparison with a reference standard in the LuMet-TCM database. Isochlorogenic acid C, taken as an example (Figure S1C), gave precursor ion at *m*/*z* 515.1189 [M-H]^−^, which generated characteristic ions at *m*/*z* 353.0874 [M-H-caffeoyl]^−^, 191.056 [M-H-2caffeoyl]^−^, 179.035 [caffeic acid-H]^−^, 173.0455 [M-H-caffeoyl-H_2_O]^−^, and 135.0453 [caffeic acid-H-CO_2_] in the MS^2^ spectrum. Compound H64 exhibited a fragmentation pathway analogous to that of isochlorogenic acid C, displaying an [M+FA-H]^−^ ion at *m*/*z* 561.1248 and was accurately identified as isochlorogenic acid A.

### 3.5. Identification and Characterization of the Absorbed Prototype Chemicals in Rat Serum

Medicated serum samples were collected and mixed after four days of continuous oral administration of TSAC in rats after the blank and drug-containing serum samples were precipitated with a mixture of methanol and acetonitrile, a UPLC-Q Exactive Orbitrap-MS.

The method was established to analyze and characterize the absorbed prototype components of TSAC. The BPI chromatograms of the medicated TSAC serum in positive and negative ion modes are shown in Figure S2A,B. The extracted-ion peaks present in the medicated serum and TSAC but not in the blank serum were identified as the prototype ingredients absorbed in the serum. In addition, the endogenous substances that simultaneously appeared in the blank and medicated sera were deduced from the extracted ion peaks because of the interference of false-positive results. The constituents of the absorbed prototype within TSAC, which were integrated and deduplicated in both positive and negative ion modes, were identified through a comparative analysis of accurate mass, retention time, and fragmented MS2 spectra between the serum constituents and those present in TSAC.

Utilizing the identification strategy previously outlined, a comprehensive analysis revealed a total of 22 absorbed prototype components in the dosed serum. This included 16 triterpenoid saponins, two steroidal saponins, and four triterpenoids, which were identified in comparison to the constituents of TSAC through the application of the extracted-ion chromatogram (EIC) function, as illustrated in Table S3 and Figure S3. The error in the identified prototype compounds was <5 ppm. Interestingly, 16 triterpenoid saponins, which account for more than half of all prototypal components, were identified in rat serum. These results confirmed that the absorbed triterpenoid saponins were the main active components of *A. chinensis*. Our findings provide indispensable support for classifying the therapeutic effects of TSAC.

### 3.6. Results of Network Pharmacology Analysis

#### 3.6.1. Targets of the Prototype Active Compounds

A comprehensive analysis utilizing the SwissTargetPrediction database identified 486 potential targets derived from 22 absorbed prototype components present in serum. Subsequently, a component-target network diagram was generated employing Cytoscape version 3.7.2, as illustrated in Figure S4A. In total, 508 nodes and 2325 edges existed in the network. The congmunoside V, elatoside D, ginsenoside Ro, and sandosaponin A with 108-degree values targeted the most proteins. The potential targets of the 22 absorbed prototype components were imported into the STRING database (https://string-db.org/, accessed on 15 July 2023) for PPI network topological analysis. The confidence level was set to >0.9, and single unconnected targets were excluded. The PPI results obtained from the STRING database were analyzed using Cytoscape version 3.7.2 for visual network analysis. The CytoHubba plug-in facilitated the extraction of the top 100 and 20 targets, determined by their degree values, as illustrated in Figure S4B,C. The larger a node, the more critical it is in the network. Therefore, these nodes may be primary targets for the therapeutic effects of TSAC.

Next, the potential targets of the 22 absorbed prototype components predicted by the SwissTargetPrediction database were used for GO and KEGG enrichment analyses using the DAVID platform. A total of 1191 GO enrichment entries were retrieved, including those for biological processes (BP, 816), cell components (CC, 122), and molecular functions (MF, 191). GO analysis indicated that these targets were associated with signal transduction, protein phosphorylation, the plasma membrane, and protein binding, as depicted in Figure S4D. Furthermore, 175 KEGG pathways were enriched, which mainly involved neuroactive ligand–receptor interactions, proteoglycans in cancer, pathways in cancer, the EGFR tyrosine kinase pathway, and the PI3K-Akt signaling pathway, as shown in Figure S4E.

#### 3.6.2. Functional Analysis of Breast Cancer-Related Genes

Utilizing “breast cancer” as a search term, a comprehensive analysis yielded a total of 1747 genes associated with breast cancer, as extracted from the CTD, DisGeNET, GeneCards, and OMIM databases, as illustrated in Figure S5A. Figure S5B delineates the top 20 signaling pathways identified, which predominantly encompass pathways related to cancer, proteoglycans in cancer, the PI3K-Akt signaling pathway, the MAPK signaling pathway, and the EGFR tyrosine kinase pathway. Furthermore, the top 100 breast cancer-related genes, including *PIK3CA*, *SRC*, *EGFR*, *AKT1*, and *STAT3*, were identified from a protein–protein interaction (PPI) network comprising the 1747 breast cancer-associated genes, based on their degree centrality, as depicted in Figure S5C. Notably, the breast cancer genes and the targets of TSAC exhibited overlapping pathways, indicating a multi-targeted mechanism of action for TSAC in the context of breast cancer.

#### 3.6.3. Intersection Analysis of Targets of Absorbed Active Components and Breast Cancer-Related Genes

Figure S6 illustrates the intersection analysis of 486 targets associated with the bioactive components in TSAC and genes related to breast cancer (Figure S6A). A total of 204 overlapping targets, along with their corresponding compounds, were utilized to construct a compound-overlapping target network. This analysis indicated that the compounds 3*β*,21*α*-Dihydroxyoleana-11,13(18)-dien-29-oic acid, caulophyllogenin, echinocystic acid, oleanolic acid, and araloside A exhibit the highest affinity for proteins associated with breast cancer, with respective target counts of 58, 55, 54, 53, and 52, as depicted in Figure S6B.The STRING platform was used to construct a PPI network of the intersections of 204 targets to analyze interactions, which involved 174 nodes and 682 edges, as shown in Figure S6C. The top 20 key targets (*SRC*, *PIK3R1*, *PIK3CA*, *STAT3*, and *EGFR*) were selected from the PPI network based on the degree values using the CytoHubba plug-in, as shown in Figure S6D.

Furthermore, the BP, CC, and MF of 204 overlapping targets were analyzed. GO enrichment analysis identified 630 BPs, 94 CCs, 147 MFs, and 172 significantly enriched KEGG pathways (*p* < 0.05). The results indicated that these targets were mainly associated with protein phosphorylation, cytosolic localization, and protein binding, as depicted in Figure S6E. As illustrated in Figure S6F, the twenty most significantly enriched signaling pathways encompassed those associated with cancer, the EGFR tyrosine kinase pathway, and the PI3K-Akt signaling pathway.

In order to investigate the critical targets contributing to the therapeutic efficacy of TSAC in breast cancer, we identified significant overlapping targets that function as both TSAC targets and genes associated with breast cancer (refer to Figure S6A). Additionally, we examined hub genes represented in the PPI network of these overlapping targets (illustrated in Figure S6D) as well as the TSAC targets (depicted in Figure S6C). The analyses indicated that *SRC*, *PIK3R1*, *PIK3CA*, *STAT3*, and *EGFR* were integral components of the hub genes within the PPI network encompassing both the overlapping and TSAC targets. These results indicated that *SRC*, *PIK3R1*, *PIK3CA, STAT3*, and *EGFR* are the core targets of TSAC in treating breast cancer.

### 3.7. Molecular Docking

The hub targets identified for *SRC*, *PIK3R1*, *PIK3CA*, *STAT3*, and *EGFR* may represent significant therapeutic targets for the treatment of breast cancer using TSAC. In this context, a molecular docking approach was employed to assess the binding energies of 22 absorbed prototype compounds about their respective targets. Among the five hub targets, Araloside A, elatoside L, and oleanolic acid-3-O-glucosyl (1-2) xylyl (1-3) glucosiduronic acid exhibited the lowest binding energy scores. Specifically, the docking interactions of elatoside L with *SRC* and *PIK3CA* yielded binding energy values of −6.9 and −9.8 kcal/mol, respectively. Additionally, the interaction between *PIK3R1* and oleanolic acid-3-O-glucosyl (1-2) xylyl (1-3) glucosiduronic acid resulted in a binding energy of −8.4 kcal/mol. The binding energies of araloside A with *STAT3* and *EGFR* were −7.9 and −6.8 kcal/mol. Additional specific docking scores of the 22 prototype components for these targets are listed in Table S4. The docking results for the hub targets and their corresponding constituents are illustrated based on the heat map in Figure S7A. Three-dimensional views of the docking patterns between the three components and the top five hub targets were constructed using PyMOL (Figure S7B–F).

### 3.8. Results of Experimental Validation In Vitro

#### 3.8.1. TSAC Inhibited the Proliferation and Colony of MCF-7 Cells

To assess the inhibitory effects of TSAC on breast cancer cells, the viability of MCF-7 cells was evaluated using the CCK-8 assay following in vitro treatment with TSAC. As illustrated in Figure S8A,B, treatment at a concentration of 0.5 mg/mL resulted in a dose- and time-dependent inhibition of MCF-7 cell growth, with viability rates recorded at 33.96 ± 2.77% and 31.40 ± 2.21% after 24 h and 48 h, respectively. Additionally, data from the colony formation assay further corroborated these findings, indicating a significant reduction in colony numbers in the TSAC-treated groups compared to the control group (Figure S8C,D).

#### 3.8.2. TSAC Inhibited Migration Rates of MCF-7 Cells

A scratch assay was conducted to assess the migratory capacity of MCF-7 cells after treatment with TSAC. As illustrated in Figure S9, after 12 h of incubation, the healing rate of MCF-7 cells exposed to TSAC at a concentration of 0.3 mg/mL diminished from 20 ± 1.00% to 6 ± 1.79% in comparison to the control group. Comparable findings were observed after 24 h of incubation. These results indicate that TSAC exerts an inhibitory effect on the migration of MCF-7 cells in a dose-dependent manner.

#### 3.8.3. Effect of TSAC on the Expression of Genes

**Figure 2:**
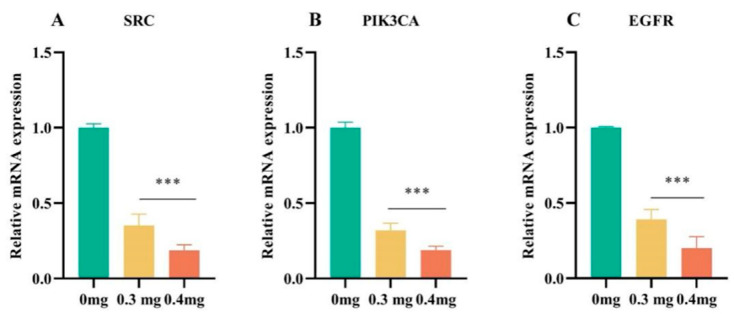
Effect of TSAC on gene expression. RT-qPCR analysis of mRNA levels of *SRC*, *PIK3CA*, and *EGFR* (**A**–**C**). Data are expressed as mean ± SD (n = 3). *** *p* < 0.001 compared with the control group.

Utilizing the findings from network pharmacology and molecular docking analyses, the hub genes were subsequently validated through RT-qPCR. As illustrated in Figure 2A–C, TSAC significantly decreased the mRNA expression levels of *SRC*, *PIK3CA*, and *EGFR* (*p* < 0.001) in comparison to the control group. These data confirm that TSAC downregulates the transcriptional activity of *SRC*, *PIK3CA*, and *EGFR*.

#### 3.8.4. TSAC Suppressed SRC, PI3K, and EGFR Expression in MCF-7 Cells

**Figure 3:**
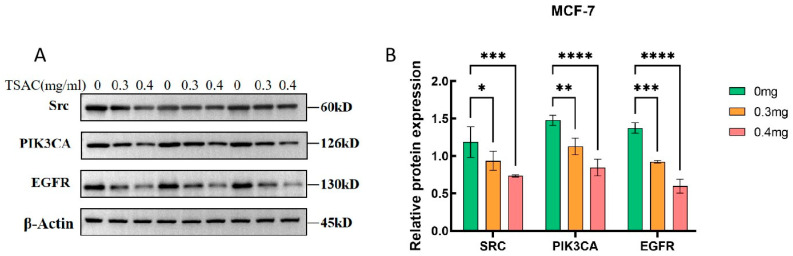
Effect of TSAC on protein expression. Expression levels of *SRC*, *PI3K*, and *EGFR* in MCF-7 cells (**A**). Changes in protein levels were presented by histograms (**B**). Data are expressed as mean ± SD (n = 3). * *p* < 0.05, ** *p* < 0.01, *** *p* < 0.001, **** *p* < 0.0001 compared with the control group.

According to the RT-qPCR results, the hub genes *SRC*, *PIK3CA*, and *EGFR* may be involved in the anti-breast cancer effects of TSAC. Next, we performed western blotting to determine the protein levels of *Src*, *PI3K,* and *EGFR*. As depicted in Figure 3A,B, identical to the RT-qPCR results, TSAC significantly decreased *Src*, *PI3K,* and *EGFR* expression levels (*p* < 0.05) compared with the control. RT-qPCR and Western blot analysis demonstrated that TSAC exerted an anti-breast cancer effect by suppressing the gene and protein levels of *Src*, *PI3K*, and *EGFR*.

## 4. Discussion

### 4.1. Introduction to TCM and Aralia in Cancer Therapy

TCM has evolved as a comprehensive system of medical practice with its theoretical framework for the diagnosis and treatment of disease. TCM can differentiate syndromes and enhances the host’s disease resistance [32]. In cancer treatment, TCM has recently attracted increasing attention owing to its advantages in improving symptoms, reducing adverse reactions to radiotherapy and chemotherapy, improving patients’ quality of life, and prolonging survival periods [33]. For instance, andrographolide, a natural diterpenoid derived from Andrographis paniculata, demonstrated enhanced therapeutic efficacy when administered in conjunction with an anti-PD-1 antibody, surpassing the effects of monotherapy in a murine xenograft model of CT26 colon cancer [34]. To date, approximately 300 chemical constituents have been isolated from the roots, root bark, stems, and leaves of *Aralia*, including triterpenoid saponins, flavonoids, polysaccharides, phenylpropanoids, and amino acids, of which nearly 200 have been isolated. Triterpenoid saponins possess significant pharmacological activities, including cardiovascular system protection, antitumor effects, hypoglycemic effects, blood lipid regulation, liver protection, nerve protection, anti-inflammatory effects, and analgesia [35,36,37,38,39,40,41,42,43,44].

### 4.2. Pharmacological Research Status and Challenges of Aralia

Upon the structural identification of the chemical constituents of *Aralia*, which were analyzed utilizing contemporary extraction and separation techniques, a scientific basis for the exploration of its pharmacological activity and underlying mechanisms of action can be established. However, the pharmacological research entities on *Aralia* are mainly alcohol extracts (or crude extracts) and total saponins; there are few pharmacological studies on monomeric compounds. This is attributed to the difficulties of phytochemical separation and purification and the fact that most monomeric compounds are usually micro-ingredients, which prevents their development from extensive pharmacological research. For instance, *Aralia* total saponins exhibited outstanding antihyperglycemic, hypolipidemic, and antioxidant activities in T2DM rats [45]. *Aralia* ethanol extract exhibits anti-breast cancer activity by activating the mitochondria-mediated apoptotic pathway with high biological safety in mice [25]. *Aralia* extracts ameliorate NAFLD by inhibiting insulin resistance by activating the Akt/GLUT4 pathway [46]. The anti-inflammatory actions of *Aralia* elata-ethyl acetate fraction may be related to the inhibition of NF-κB activation in LPS-stimulated macrophages [47]. *Aralia* total aralosides can protect the liver against non-alcoholic steatohepatitis by regulating IRE1α-mediated JNK and NF-κB signal pathways [39]. *Aralia* total saponins have a positive inotropic effect on canine myocardium and isolated rat cardiomyocytes [48].

### 4.3. Clinical Applications and Resource Sustainability Concerns

To date, 12 types of Chinese patent medicines consisting of *Aralia* have been developed and marketed in China following approval by the National Medical Products Administration. These formulations have demonstrated therapeutic efficacy when administered orally. Notable examples include the Longya Gantai Capsule, which serves as an adjunctive treatment for acute and chronic hepatitis characterized by elevated transaminase levels; the Pingxuan Capsule, indicated for symptoms such as dizziness, palpitations, tinnitus, insomnia, and discomfort in the lumbar and knee regions; the Hewei Jiangni Capsule, utilized for chronic superficial gastritis and chronic atrophic gastritis; the Baowei Capsule, prescribed for epigastric pain as well as gastric and duodenal ulcers; and the Congmu Weitong granules, which are effective for gastritis, gastric and duodenal pain, and occult bleeding. Although these drugs have been used for many years, no systematic and comprehensive investigation has been conducted on the absorbed active ingredients of *Aralia* into the blood after oral administration. Therefore, research on the bioactive ingredients of *Aralia* based on serum pharmacochemistry will help to clarify the underlying mechanism of action.

*A. chinensis* has a wide range of uses as a regular medicine for ethnic minorities in Guizhou Province. For example, the Miao people of Guizhou Province use the root bark to treat rheumatoid arthritis, gastric and duodenal ulcers, trauma, and snakebites. As a plant used for both medicinal and edible purposes, the demand for the tender buds and leaves of *Aralia* has increased considerably. However, owing to the destruction of the ecological environment and the influence of unreasonable collection, wild *Aralia* resources have rapidly declined. If adequate protective measures are not implemented, the unrestricted extraction of buds and roots will inevitably lead to resource depletion, causing the rapid diminishment and potential exhaustion of reserves. Therefore, it is imperative to implement policies to ensure the continuous proliferation of natural resources. Furthermore, the sustainable utilization of these resources can be achieved through regulated extraction and artificial cultivation.

### 4.4. Chemical Profiling of TSAC and Identification of Absorbed Components

Based on high-resolution UPLC-Q Exactive Orbitrap-MS technology, we identified 38 triterpenoid saponins, three steroidal saponins, and eight triterpenoids from TSAC by comparing the standard library (LuMet-TCM database) and literature information, which can provide a scientific basis for the study of breast cancer treatment of *A. chinensis*. In addition, we identified various other microcomponents, such as phenylpropanoids, terpenoids, saccharides, phenols, amino acids and peptides, carboxylic acids and derivatives, organic acids and their derivatives, and alkaloids, which provided new insights for exploring the pharmacodynamics and key pharmacological mechanisms of *A. chinensis*. Moreover, we probed the composition of the absorbed prototype components of the TSAC. A total of 22 prototype components, including 16 triterpenoid saponins, four triterpenoids, and two steroidal saponins, were identified, of which triterpenoid saponins and aglycones were dominant. Several of these compounds exhibit diverse bioactivities. For example, chikusetsusaponin Ⅳa exerts a therapeutic effect on T2DM by regulating glucose uptake and fatty acid oxidation in rats [49]. Calenduloside E can ameliorate non-alcoholic fatty liver disease by inhibiting pyroptosis signaling pathways [50]. Araloside A shows pro-apoptotic and anti-inflammatory effects in rheumatoid arthritis fibroblast-like synoviocytes by inhibiting the nuclear factor kappa B pathway [51]. Araloside C lessened atherosclerosis by modulating macrophage polarization in RAW264.7 macrophages via Sirt1-mediated autophagy [52].

### 4.5. Serum Pharmacochemistry: Rationale for Identifying Bioactive Compounds

Bioactive compounds derived from Traditional Chinese Medicine (TCM) hold considerable importance for understanding their therapeutic mechanisms. Nevertheless, the process of identifying active compounds in TCM through traditional methodologies, including extraction, separation, and structural characterization, is labor-intensive and time-consuming. Furthermore, it is important to note that not all constituents identified within TCM possess therapeutic properties. Conversely, the components that are absorbed into the bloodstream following the oral administration of TCM are more likely to be the effective agents responsible for therapeutic outcomes [53]. UPLC-Q Exactive Orbitrap-MS technology based on serum pharmacochemistry can quickly identify absorbed components following oral delivery [54,55,56].

TCM comprises a complex system reflected in the diversity of chemical components and biological reactions in the body. This diversity indicates no simple interaction between components, which should include the coordination and unity of synergy, addition, and antagonism. The modes of action of multiple components and targets are not simple. Close synergistic and antagonistic interactions were observed between these components. Saponins with diverse skeletal structures in *A. chinensis* can be investigated collectively or individually to evaluate their synergistic or antagonistic interactions using genomics, proteomics, metabolomics, and other techniques in future work.

### 4.6. Network Pharmacology and Mechanism Elucidation of TSAC in Breast Cancer

Network pharmacology based on systems biology is widely used to predict pharmacological action mechanisms and is a powerful approach for studying TCM with complex components and multiple targets [57]. Serum pharmacochemistry combined with network pharmacology analysis is a reliable and rapid strategy for identifying the active components absorbed into the blood following oral administration of TCM compounds and screening the core targets of disease treatment [58]. Prior research has demonstrated that the candidate compounds detected in the serum of rats treated with traditional Chinese medicine (TCM) can be classified as bioactive compounds suitable for network pharmacology analysis. For example, following the oral administration of Qing-Fei-Pai-Du decoction, twelve active compounds were identified in the serum of mice. Furthermore, a total of fifty-five significant targets were identified from these twelve absorbed compounds, which are relevant to the treatment of COVID-19 with Qing-Fei-Pai-Du decoction [59].

Genes implicated in the pathogenic mechanisms of breast cancer were found to be associated with cancer-related pathways, proteoglycans in cancer, and the PI3K-Akt signaling pathway. Additionally, the targets of the 22 compounds predominantly encompassed neuroactive ligand–receptor interactions, cancer pathways, and proteoglycans in cancer, all of which are pertinent to breast cancer. In this investigation, the correlated targets of the 22 components, recognized as potential bioactive compounds in the context of breast cancer, were analyzed through network pharmacology methodologies. The 486 targets of the 22 compounds and 1747 breast cancer-related genes were comprehensively explored. The cancer pathways, proteoglycans in the tumor microenvironment, EGFR tyrosine kinase pathway, and PI3K-Akt signaling pathway may correlate with the therapeutic effect of TSAC in treating breast cancer. Proteoglycans, characterized by a protein core to which long glycosaminoglycan (GAG) chains are covalently linked, play a significant role in various molecular and cellular processes associated with the advancement of solid tumors. They fulfill multiple functions within the context of cancer development and progression [60,61]. KEGG analysis indicated that TSAC may interfere with proteoglycans, thereby reducing or blocking breast cancer metastasis. Overexpression of EGFR, which belongs to the ERBB family of RTKs, has been observed in different types of cancers, including breast [62,63]. Increased levels of RTKs are associated with breast cancer progression [64]. The EGFR tyrosine kinase pathway is closely associated with various tumors. This pathway is a crucial mediator that regulates the proliferation, metabolism, and apoptosis of tumor cells. Epidermal growth factor receptor (*EGFR*) has been recognized as a pivotal target in the development of anti-tumor pharmacological agents [65,66,67]. The phosphoinositide 3-kinase (*PI3K*)-Akt signaling pathway is integral to numerous cellular processes that are dysregulated in cancer, thereby facilitating tumor initiation and progression. It is a hallmark of tumor cell survival, metastasis, and metabolism [68]. Multiple growth factor receptors, including the *EGFR* and *HER2*, influence the activation of *PI3K*.

### 4.7. Proposed Mechanism of Action and Experimental Validation

Drawing from the PPI and enhanced KEGG analyses, we hypothesize that the therapeutic efficacy of TSAC in breast cancer may be linked to its inhibitory effects on the EGFR/PI3K/Akt signaling pathways. Furthermore, validated molecular docking studies demonstrated that active compounds, including araloside A and elatoside L, can bind directly to *EGFR* and *PIK3CA*. According to the findings from predictive network pharmacology and molecular docking analyses, TSAC appears to inhibit the proliferation and migration of MCF-7 cells. The RT-qPCR results revealed that the expression of *SRC*, *PIK3CA* and *EGFR* decreased dose-dependently after TSAC treatment. Similarly, the protein levels of *Src*, *PI3K*, and *EGFR* markedly decreased after TSAC treatment.

### 4.8. Conclusion and Future Perspectives

In conclusion, our findings provide valuable insights into potential novel targets and signaling pathways of TSAC for breast cancer treatment. The detailed mechanism of action of TSAC on EGFR/PI3K/AKt signaling in breast cancer requires further investigation.

## 5. Conclusions

In conclusion, this study systematically explored the anti-breast cancer mechanism of total saponins from *Aralia chinensis* L. (TSAC) through integrated phytochemical and pharmacological approaches. Using UHPLC-MS, 49 components in TSAC were identified, including 38 triterpenoid/steroidal saponins, three steroidal saponins, and eight triterpenoids, with 22 blood-absorbed prototype components confirmed as pharmacodynamically active constituents. Network pharmacology combined with experimental validation revealed that TSAC exerts anti-breast cancer effects by multi-target inhibition of the *Src/PI3K/EGFR* signaling axis: molecular docking experiments confirmed that active components such as araloside A and elatoside L have high-affinity binding to key targets *SRC*, *PIK3CA*, and *EGFR*; in vitro experiments further verified that TSAC downregulates the expression of *Src*, *PI3K*, and *EGFR* at both gene and protein levels in a dose-dependent manner. These findings provide a solid pharmacological basis for the development of *Aralia chinensis*-derived anti-breast cancer agents. For future research, it is recommended to purify dominant active components for in vivo efficacy and toxicity evaluation, validate the multi-target mechanism in patient-derived xenograft (PDX) models, and explore synergistic effects with standard chemotherapeutic drugs (e.g., paclitaxel) to lay a foundation for clinical translation.

## Figures and Tables

**Table 1:** Sequences of PCR primers for target gene detection.

Gene	Sequence (5’–3’)
*β-actin*	Forward: TGAGCTGCGTTTTACACCCT
	Reverse: AAGTCAGTGTACAGGCCAGC
*SRC*	Forward: TGGTTTCAGAGGAGCCCATTTAC
	Reverse: CACTTTGCACACCAGGTTCTCTC
*PIK3CA*	Forward: TATTGTCGTGCATGTGGGATGTA
	Reverse: GCAGGGTTTAGAGGAGACAGAAA
*EGFR*	Forward: CTGGGTGCGGAAGAGAAAGAATA
	Reverse: CCAAAGGTCATCAACTCCCAAAC

## Data Availability

The data are included in the article. Further inquiries can be directed to the corresponding authors.

## Supplementary Materials

The following supporting information can be downloaded from: https://www.mdpi.com/article/10.3390/cimb47080662/s1.
