# Supplementary material for: Unlocking the Anti-Breast Cancer Potential of Aralia chinensis L
Source: Curr Issues Mol Biol. 2025 Aug 16;47(8):662. doi: 10.3390/cimb47080662 (PMC12384973; doi:10.3390/cimb47080662)
Supplement: Supplementary file 1 [file cimb-47-00662-s001.zip › Table S1.pdf]

Table S1. Identification of the chemical constituents of TSAC using UPLC-Q Exactive Orbitrap-MS.

No.	Retention time	Formula	Identification	theoretical mass	Experimental mass	Adduct ion	Error (ppm)	Fragment Ions
triterpenoid saponins								
H1	5.28	$C_{42}H_{68}O_{15}$	Congmuyanoside I	857.4540	857.4538	M+FA-H	-0.24	487.3436(1.52%), 649.3997(4.22%), 811.4457(100.00%), 857.4573(10.67%)
H2	5.58	$C_{36}H_{58}O_{11}$	Quercilicoside A*	665.3907	665.3905	M-H	-0.23	503.1539(0.76%), 665.3903(100.00%)
H3	5.70	$C_{65}H_{104}O_{33}$	Elatoside L	1435.6352	1435.6348	M+Na	-0.29	439.3569(0.60%), 509.1472(30.83%), 965.5067(21.42%), 1435.6321(100.00%)
H4	5.96	$C_{47}H_{74}O_{19}$	Armatoside B	941.4751	941.4744	M-H	-0.78	455.3535(1.37%), 569.3842(0.35%), 587.387(0.11%), 647.3835(0.30%), 779.4205(1.49%), 809.4362(0.69%), 941.4738(100.00%)
H5	6.17	$C_{42}H_{66}O_{15}$	Elatoside H	809.4329	809.4325	M-H	-0.45	471.3478(0.29%), 587.3606(0.16%), 647.382(7.15%), 809.4324(100.00%)
H6	6.19	$C_{36}H_{62}O_{9}$	Ginsenoside F1	621.4361	621.4359	M+H-H_2O	-0.31	423.3605(37.29%), 441.3716(100.00%), 459.3825(86.02%), 621.4294(6.97%)
H7	6.23	$C_{59}H_{94}O_{28}$	Congmunoside XI	1295.5914	1295.5906	M+FA-H	-0.64	455.3531(20.33%), 613.3721(2.81%), 731.4386(7.00%), 925.4793(3.48%), 1087.5323(100.00%), 1117.5505(5.71%), 1249.5842(26.26%)
H8	6.40	$C_{42}H_{68}O_{14}$	Deoxytibesaikosaponin V*	841.4591	841.4585	M+FA-H	-0.76	453.3395(0.31%), 471.3487(0.86%), 633.4053(7.05%), 795.4507(100.00%), 841.4553(16.16%)
H9	6.41	$C_{36}H_{56}O_{11}$	Elatoside G	647.3790	647.3791	M+H-H_2O	0.20	425.3406(1.75%), 435.3247(15.79%), 453.3354(100.00%), 471.3449(6.99%),

								629.3668(3.04%), 647.3785(24.65%)
H10	6.55	$C_{42}H_{68}O_{15}$	Tibesaikosaponin V*	857.4540	857.4534	M+FA-H	-0.74	489.5708(0.27%), 517.1129(0.73%), 649.3905(0.89%), 811.4482(100.00%), 857.4520(12.30%)
H11	6.59	$C_{42}H_{72}O_{14}$	Ginsenoside Rf*	845.4904	845.4898	M+FA-H	-0.76	410.1240(0.34%), 453.7000(0.35%), 490.8191(0.36%), 657.9109(0.32%), 723.4998(0.32%), 735.4373(0.33%), 799.3312(100.00%), 845.4923(18.66%)
H12	6.78	$C_{54}H_{88}O_{23}$	Congmunoside V	1149.5698	1149.5721	M+FA-H	2.04	455.3545(23.06%), 617.4058(14.03%), 779.4579(11.14%), 941.5101(100.00%), 1103.5632(23.21%), 1149.5645(17.02%)
H13	6.78	$C_{53}H_{84}O_{23}$	Elatoside C	1133.5385	1133.5384	M+FA-H	-0.12	455.3532(32.88%), 569.3851(12.48%), 613.3774(1.90%), 731.4396(2.32%), 793.4384(5.82%), 925.4767(11.59%), 955.4897(61.87%),1087.5258(100.00%), 1133.5414(16.80%)
H14	6.78	$C_{48}H_{76}O_{19}$	Elatoside I	979.4873	979.4870	M+Na	-0.31	431.119(0.49%), 461.1211(0.17%), 523.2016(26.92%), 641.4056(0.19%), 751.2085(0.07%), 761.5175(0.08%), 791.1583(0.07%), 803.4448(0.21%), 810.8768(0.07%), 819.6225(0.06%), 911.7361(0.06%), 914.277(0.07%), 979.4860(100.00%)
H15	6.82	$C_{54}H_{86}O_{24}$	Hemsloside G2	1117.5436	1117.5427	M-H	-0.83	119.0351(0.20%), 179.0560(0.13%), 455.3523(6.44%), 569.3905(0.32%), 613.3730(0.27%), 631.3842(0.15%),

								731.4392(2.23%), 793.4394(0.87%), 955.4874(7.23%), 1117.5419(100.00%)
H16	6.85	$C_{54}H_{86}O_{24}$	Elatoside D	1141.5401	1141.5399	M+Na	-0.17	461.1254(0.02%), 509.1481(0.02%), 523.1258(50.37%), 541.1401(0.46%), 641.4012(1.61%), 969.4690(0.02%), 1141.5374(100.00%)
H17	7.02	$C_{53}H_{84}O_{23}$	Araloside C*	1087.5330	1087.5320	M-H	-0.94	455.3529(5.70%), 613.3754(0.27%), 731.4390(12.29%), 763.4275(0.45%), 793.4380(0.49%), 925.4767(5.27%), 955.4816(0.58%), 1087.1555(100.00%)
H18	7.26	$C_{48}H_{76}O_{19}$	SpinasaponinA 28-O-glucoside	979.4873	979.4870	M+Na	-0.29	121.1015(0.28%), 181.0119(0.22%), 379.0838(2.36%), 523.1293(1.32%), 623.3983(0.14%), 641.4013(4.80%), 684.3849(0.14%), 803.4539(0.04%), 979.4858(100.00%)
H19	7.26	$C_{48}H_{76}O_{19}$	Sandosaponin A	955.4908	955.49	M-H	-0.81	455.3532(3.25%), 569.3830(9.45%), 587.3931(1.51%), 613.3723(2.62%), 631.3846(1.29%), 731.4378(3.62%), 793.4350(10.30%), 955.4884(100.00%)
H20	7.28	$C_{48}H_{76}O_{19}$	Ginsenoside Ro*	974.5319	974.5317	$M+NH_4$	-0.18	191.1791(18.26%), 321.0806(14.02%), 339.0915(25.27%), 393.3505(5.43%), 439.3561(100.00%), 501.1448(2.43%), 795.4465(1.59%), 974.5319(2.18%)
H21	7.59	$C_{47}H_{74}O_{18}$	Araloside A*	944.5213	944.5216	$M+NH_4$	0.27	121.1011(2.23%), 201.1639(7.89%), 439.3562(100.00%), 471.1333(1.72%), 944.5149(3.49%)

H22	7.73	$C_{53}H_{86}O_{23}$	Aralia-saponin III	1071.5382	1071.5376	M-H_2O-H	-0.53	455.3530(6.41%), 569.3848(12.44%), 731.4369(15.12%), 925.4736(4.68%), 1071.5348(100.00%)
H23	7.86	$C_{42}H_{66}O_{14}$	Chikusetsusaponin Ⅳ a*	793.4379	793.4375	M-H	-0.54	455.3531(0.19%), 569.3846(13.24%), 613.3764(2.06%), 631.3835(17.02%), 793.4346(100.00%)
H24	8.19	$C_{36}H_{56}O_{10}$	Potentillanoside A*	649.3947	649.3942	M+H	-0.70	191.1793(25.35%), 437.3405(100.00%), 455.3479(23.44%), 649.3923(39.83%)
H25	8.57	$C_{48}H_{78}O_{18}$	Congmuyanoside F	987.5171	987.5167	M+FA-H	-0.38	455.3528(100.00%), 647.3805(0.78%), 779.4538(13.28%), 941.4982(45.72%), 987.5134(33.39%)
H26	8.85	$C_{48}H_{78}O_{18}$	Silphioside E	987.5170	987.5175	M+FA-H	0.48	455.3535(23.90%), 617.4041(6.09%), 779.4579(10.54%), 925.4819(4.23%), 941.5082(100.00%), 987.5148(18.70%)
H27	8.89	$C_{48}H_{78}O_{19}$	Congmuyenoside I	939.4959	939.4961	M-H_2O-H	0.22	455.3551(5.28%), 569.3849(16.15%), 575.5934(0.53%), 595.3659(4.63%), 648.5557(0.42%), 675.7114(0.42%), 694.1721(0.56%), 731.4381(13.59%), 775.4194(0.80%), 925.4836(0.77%), 939.4929(100.00%)
H28	8.89	$C_{36}H_{58}O_{9}$	Pomolic acid β-D-glucopyranosyl ester*	679.4063	679.4058	M+FA-H	-0.78	471.3475(100.00%), 633.3970(3.86%), 679.4081(39.82%)
H29	8.97	$C_{48}H_{76}O_{19}$	Elatoside B	979.4873	979.4868	M+Na	-0.49	379.0860(0.44%), 469.5670(0.02%), 495.1330(0.16%), 523.1260(38.80%), 541.1386(0.12%), 587.9670(0.03%),

								603.0250(0.02%), 656.9134(0.03%), 663.9720(0.03%), 684.3849(0.08%), 738.3025(0.02%), 805.2997(0.03%), 811.8661(0.03%), 979.4854(100.00%)
H30	9.12	$C_{47}H_{74}O_{18}$	Oleanolic acid-3-O-glucosyl(1-2)xylyl(1-3) glucosiduronic acid*	971.4857	971.4846	M+FA-H	-1.15	455.3506(2.05%), 569.3857(6.96%), 655.6432(0.75%), 731.4400(2.08%), 801.5801(0.70%), 925.4788(100.00%), 971.4691(0.82%)
H31	9.19	$C_{47}H_{74}O_{18}$	Araloside H	944.5214	944.521	$M+NH_4$	-0.38	121.1016(3.81%), 201.1631(8.27%), 339.0914(15.12%), 439.3562(100.00%), 471.1338(8.40%), 489.1425(1.49%), 795.4419(2.57%)
H32	9.21	$C_{47}H_{74}O_{18}$	Pseudoginsenoside RT1	925.4802	925.4795	M-H	-0.81	455.3531(7.00%), 569.3841(8.44%), 587.3932(0.49%), 613.3754(0.19%), 631.3887(0.22%), 731.4368(6.27%), 763.4348(2.41%), 793.4395(0.31%), 925.4799(100.00%)
H33	9.23	$C_{42}H_{66}O_{14}$	Zingibroside R1*	812.4791	812.4784	$M+NH_4$	-0.82	184.0730(100.00%), 191.1788(11.33%), 339.0911(12.50%), 393.3514(3.03%), 439.3564(55.18%), 484.5266(0.35%), 628.9764(0.40%), 812.4580(11.99%)
H34	9.25	$C_{36}H_{58}O_9$	Hederagenin 28-D-glucoside*	679.4064	679.4057	M+FA-H	-0.97	471.3475(100.00%), 633.4026(4.73%), 679.4084(36.51%)
H35	9.49	$C_{42}H_{68}O_{13}$	Randianin	825.4643	825.4640	M+FA-H	-0.31	455.3528(100.00%), 617.4028(0.60%), 779.4581(64.11%), 825.4543(11.80%)
H36	9.91	$C_{41}H_{66}O_{14}$	Congmuyanoside A	763.4274	763.4270	$M-H_2O-H$	-0.51	455.3518(3.56%), 587.3929(0.13%),

								631.3862(0.75%), 763.4263(100.00%)
H37	10.19	$C_{36}H_{56}O_{9}$	Calenduloside E*	631.3851	631.3846	M-H	-0.81	455.3524(7.38%), 509.3621(1.97%), 511.3411(0.29%), 555.3693(2.34%), 571.3682(0.04%), 613.374(0.17%), 631.3838(100.00%)
H38	10.58	$C_{36}H_{58}O_{8}$	Oleanolic acid β-D-glucopyranosyl ester	663.4114	663.4108	M+FA-H	-0.91	455.3527(100.00%), 617.4039(6.29%), 663.4113(43.97%)
steroid saponins								
H39	6.04	$C_{47}H_{74}O_{19}$	Deslanoside*	987.4808	987.4789	M+FA-H	-1.88	85.0296(12.25%), 119.0351(10.26%), 487.3445(1.15%), 941.4976(49.38%), 987.4807(100.00%)
H40	6.74	$C_{41}H_{64}O_{14}$	Digoxin*	825.4279	825.4270	M+FA-H	-1.03	101.0246(14.06%), 119.0350(6.14%), 779.4222(13.21%), 825.4262(100.00%)
H41	7.22	$C_{41}H_{64}O_{13}$	Ophiopogonin A	809.4329	809.4321	M+FA-H	-1.00	455.3539(0.56%), 471.3492(7.91%), 569.3846(3.63%), 585.3803(0.47%), 587.3945(0.36%), 601.3698(7.91%), 763.4273(28.70%), 809.4326(100.00%)
triterpenoids								
H42	6.25	$C_{30}H_{48}O_{5}$	Caulophyllogenin	471.3469	471.3470	M+H-H_2O	0.24	391.3342(2.76%), 435.3241(9.96%), 453.3353(63.16%), 471.3458(100.00%)
H43	6.25	$C_{30}H_{48}O_{2}$	Betulinaldehyde*	441.3727	441.3742	M+H	3.35	233.1900(9.20%), 405.3508(11.31%), 423.3658(100.00%)
H44	6.41	$C_{30}H_{46}O_{4}$	3beta,21alpha-Dihydroxyoleana-11,13(18)-dien-29-oic acid	453.3364	453.3364	M+H-H_2O	0.06	389.3192(3.25%), 407.3303(7.88%), 435.3227(17.82%), 453.3338(100.00%)

H45	7.01	$C_{30}H_{48}O_3$	Oleanolic acid	457.3676	457.3674	M+H	-0.46	393.3501(7.31%), 411.3612(100.00%), 439.3563(54.60%), 457.3666(41.68)
H46	7.86	$C_{30}H_{48}O_3$	10-hydroxy-2,2,6a,6b,9,9,12a-heptamethyl-3,4,4a,5,6,6a,7,8,8a,10,11,12,13,14b-tetradecahydro-1H-picene-4-carboxylic acid	439.3571	439.3571	M+H-H_2O	0.02	393.3505(6.09%), 421.3463(0.97%), 439.3561(100.00%)
H47	8.17	$C_{30}H_{48}O_4$	Echinocystic acid	455.3520	455.3519	M+H-H_2O	-0.25	391.3354(3.73%), 409.3452(3.39%), 437.3409(57.44%), 455.3510(100.00%)
H48	9.46	$C_{30}H_{48}O_5$	Arjunolic acid*	489.3575	489.3575	M+H	0.02	425.3395(13.90%), 443.3524(7.47%), 453.3368(70.79%), 471.3464(16.07%), 489.3576(100.00%)
H49	10.96	$C_{30}H_{48}O_4$	Hederagenin*	473.3626	473.3623	M+H	-0.57	409.3492(1.44%), 437.3405(81.80%), 455.3508(24.67%), 473.3616(100.00%)

*Identified by comparison with reference standards in the LuMet-TCM database.
