# Supplementary material for: Unlocking the Anti-Breast Cancer Potential of Aralia chinensis L
Source: Curr Issues Mol Biol. 2025 Aug 16;47(8):662. doi: 10.3390/cimb47080662 (PMC12384973; doi:10.3390/cimb47080662)
Supplement: Supplementary file 1 [file cimb-47-00662-s001.zip › Table S2.pdf]

Table S2. other compounds identified in TSAC with micro percentages

No.	Retention time	Formula	Identification	theoretical mass	Experimental mass	Adduct ion	Error (ppm)	Fragment Ions
**terpenes**								
H50	4.44	$C_{19}H_{30}O_8$	Roseoside	431.1924	431.1920	M+FA-H	-0.82	71.0139(13.99%), 89.0245(28.70%), 101.0245(11.06%), 113.0246(9.19%), 119.0346(9.76%), 161.0462(7.91%), 179.0561(18.11%), 179.1074(8.28%), 205.1234(16.52%), 431.1929(100.00%)
H51	5.35	$C_{22}H_{38}O_{11}$	Nuciferoside	523.2397	523.2394	M+FA-H	-0.49	135.0450(0.19%), 315.1812(5.68%), 477.2333(100.00%), 523.2379(15.09%)
H52	6.25	$C_{30}H_{48}O_2$	Betulinaldehyde	441.3727	441.3742	M+H	-0.10	217.1943(5.34%), 233.1900(9.20%), 405.3508(11.31), 423.3658(100.00%)
H53	9.26	$C_{10}H_{18}O$	Nerol	137.1324	137.1324	M+H-H2O	-0.26	81.0703(100.00%), 95.0858(76.80%), 109.1014(5.09%), 137.1321(23.37%)
H54	9.50	$C_{10}H_{18}O$	Isoborneol	137.1325	137.1325	M+H-H2O	0.14	81.0703(00.00%), 91.0545(26.10%), 95.0858(76.83%), 109.1013(5.43%), 110.0602(5.26%), 137.1321(24.11%)

**phenylpropanoids**								
H55	3.75	$C_{16}H_{18}O_9$	Neochlorogenic acid	353.0878	353.0876	M-H	-0.53	135.0452(39.63%), 179.0349(55.56%), 191.0560(100.00%), 353.0876(35.47%)
H56	3.99	$C_{15}H_{16}O_9$	Daphnin	339.0721	339.0718	M-H	-0.92	177.0194(100.00%), 339.0723(21.60%)
H57	4.01	$C_{14}H_{16}O_9$	Bergenin	327.0721	327.0720	M-H	-0.44	71.0140(7.91%), 89.0245(12.97%), 101.0245(6.57%), 146.9386(5.75%), 147.0453(100.00%), 192.0063(14.16%), 193.0141(6.32,%), 207.0294(5.03%), 234.0168(8.07%), 327.0725(11.02%)
H58	4.16	$C_{16}H_{18}O_9$	Chlorogenic acid	353.0878	353.0877	M-H	-0.38	135.0453(9.24%), 173.0455(16.86%), 179.0350(11.93%), 191.0560(100.00%), 353.0872(11.40%)
H59	4.31	$C_9H_{10}O_4$	Dihydrocaffeic acid	181.0506	181.0507	M-H	0.51	109.0659(10.60%), 112.9858(61.28%), 119.0503(10.36%), 121.0297(15.85%), 135.0453(15.69%), 136.0486(17.98%), 137.0609(100.00%), 166.0271(5.21%), 180.0389(5.46%), 181.0507(93.09%)
H60	4.40	$C_9H_6O_4$	Esculetin	179.0339	179.0341	M+H	1.03	133.0282(7.84%), 147.0437(31.37%), 161.0594(16.28%), 179.0335 (100.00%)
H61	4.62	$C_{17}H_{20}O_9$	4-Feruloylquinic	369.1180	369.1185	M+H	3.35	145.0281(20.74%), 163.0386(10.85), 177.0542(100.00%)

			acid					
H62	4.64	$C_{17}H_{20}O_9$	3-Feruloylquinic acid	367.1035	367.1032	M-H	-0.7	93.0347(27.03%), 173.0455(30.81%), 191.0561(100.00%), 367.1030(24.72%)
H63	4.98	$C_{20}H_{28}O_{10}$	Rosarin	473.1664	473.1664	M+FA-H	-1.03	181.1238(11.41%), 191.0556(9.91%), 221.1158(7.39%), 247.0948(100.00%), 263.1266(17.17%), 427.1568(70.65%)
H64	5.06	$C_{25}H_{24}O_{12}$	Isochlorogenic acid A	561.1249	561.1248	M+FA-H	-0.25	179.0351(62.51%), 191.0562(100.00%), 353.0875(46.06%), 515.1194(19.64%)
H65	5.11	$C_{26}H_{32}O_{11}$	Pinoresinol 4-O-beta-D-glucopyranoside	565.1927	565.1921	M+FA-H	-0.07	309.0766(100.00%), 323.0913(25.87%), 324.0997(92.45%), 339.1236(85.81%), 357.1342(16.26%), 519.1837(7.27%), 565.1937(5.12%)
H66	5.11	$C_{28}H_{36}O_{13}$	Acanthoside B	603.2048	603.2048	M+Na	-0.07	1603.2012(100.00%)
H67	5.13	$C_{25}H_{24}O_{12}$	Isochlorogenic acid C	515.1195	515.1191	M-H	-0.69	135.0453(35.35%), 155.035(6.67%), 161.0244(7.90%), 179.035(100.00%), 353.0874(72.20%), 515.1189(34.07%)
H68	5.20	$C_9H_{10}O_3$	Apocynin	167.0703	167.0704	M+H	1.03	133.0282(6.02%), 147.0437(5.14%), 151.0386(11.01%), 161.0594(6.44%), 179.0335(100.00%)

H69	5.55	$C_{26}H_{26}O_{12}$	4,5-Di-O-caffeoylquinic acid methyl ester	531.1497	531.1497	M+H	0.04	145.0281(26.24%), 163.0386(36.95%), 177.0543(100.00), 513.1378(14.49%)
H70	5.57	$C_{11}H_{12}O_4$	Sinapaldehyde	209.0808	209.0809	M+H	0.32	163.0744(7.84%), 177.0543(100.00%), 191.0702(40.19%), 191.1426(27.90%), 209.0801(84.13%)
H71	5.57	$C_{10}H_{10}O_3$	Coniferaldehyde	179.0703	179.0703	M+H	0.19	119.0491(31.43%), 133.0647(17.84%), 147.0437(100.00%), 161.0593(52.32%), 179.0703(55.22%)
H72	6.28	$C_{22}H_{26}O_8$	Syringaresinol	419.1701	419.1701	M+H-H2O	0.02	330.1090(100.00%), 351.1222(11.33%), 368.1231(9.83%), 383.1485(20.63%), 401.1602(48.62%)
H73	6.47	$C_{11}H_{12}O_4$	Ethyl Caffeic acid	207.0662	207.0662	M-H	1.58	73.0296(9.58%), 135.0452(15.62%), 161.0253(10.77%), 161.0455(25.79%), 179.0350(19.71%), 207.0662(100.00%)
H74	6.49	$C_{20}H_{20}O_6$	(+)-Balanophonin	357.1332	357.1330	M+H	-0.66	307.0957(74.78%), 311.1999, 321.1117(15.38%), 339.1221(17.39%), 357.1326(100.00%)
**phenols**								
H75	4.40	$C_8H_8O_3$	3',4'-Dihydroxyacet	151.0401	151.0402	M-H	0.76	151.0401(100.00%)

			ophenone					
H76	5.02	$C_8H_8O_3$	Isovanillin	153.0546	153.0547	M+H	0.46	111.0442(100.00%), 125.0597(59.89%), 135.1166(7.33%), 153.0546(26.31%)
H77	5.11	$C_9H_{10}O_4$	Syringaldehyde	183.0652	183.0653	M+H	0.6	113.9637(27.57%), 123.0440(100.00%), 131.9741(19.20%), 159.9689(13.44%), 183.0650(10.26%)
H78	8.39	$C_9H_{10}O_2$	2'-Hydroxy-4'-methylacetophenone	133.0648	133.0649	M+H-H2O	0.64	69.0704(6.01%), 86.0968(100.00%), 109.5152(32.94%), 117.9595(5.56%), 133.0646(65.31%)
H79	8.83	$C_{10}H_{18}O$	Alpha-Terpineol	137.1325	137.1325	M+H-H2O	0.76	81.0703(100.00%), 95.0858(1.37%), 109.0649(4.65%), 137.0455(94.00%)
**alkaloids**								
H80	4.29	$C_7H_7NO_3$	Methyl 5-hydroxypyridine-2-carboxylate	154.0499	154.0501	M+H	1.22	90.9480(5.51%), 100.5103(5.30%), 107.0857(5.19%), 113.9637(28.85%), 131.9742(17.98%), 140.0340(100.00%), 154.0502(11.76%)
H81	5.41	$C_8H_{12}N_2O_6$	Kifunensine	277.0679	277.0667	M+FA-H	-4.38	87.0088(7.40%), 2080.9172(6.09%), 209.0794(100.00%)
H82	6.32	$C_{17}H_{12}N_2O_4$	Flazin	309.0869	309.0870	M+H	0.18	235.0858(1.99%), 263.0809(47.74%), 281.0915(14.84%), 291.1616(10.00%), 309.0862(100.00%)

**saccharides**								
H83	0.67	$C_5H_{10}O_4$	Deoxyribose	117.0547	117.0549	M+H-H2O	1.81	72.0814(100.00%), 88.0760(6.75%), 99.0443(15.84%), 117.0548(10.86%)
H84	0.88	$C_6H_{12}O_6$	Glucose	225.0616	225.0616	M+FA-H	0.14	89.0245(100.00%), 119.0350(18.09%), 178.8804(20.40%)
H85	0.92	$C_{18}H_{32}O_{16}$	Manninotriose	549.1672	549.1673	M-H	0.15	89.0245(100.00%), 179.0564(49.97%), 341.1077(14.18%), 383.1186(48.02%), 503.1630(49.50%)
H86	0.92	$C_{12}H_{22}O_{11}$	Cellobiose	387.1144	387.1143	M+FA-H	-0.37	89.0245(100.00%), 161.0457(9.87%), 179.0564(26.09%), 221.0666(8.58%), 341.1083(61.19%)
H87	0.94	$C_6H_{10}O_6$	1,4-D-Gulonolactone	177.0404	177.0406	M-H	0.99	87.0088(100.00%), 99.0089(31.21%), 129.0195(67.32%), 162.0326(21.55%), 177.0188(36.98%)
**carboxylic acids and organic acids**								
H88	0.97	$C_5H_6O_5$	Oxoglutaric acid	191.0196	191.0199	M+FA-H	1.31	87.0088(34.25%), 102.9489(7.22%), 111.0087(100.00%), 124.0073(14.66%), 129.0193(9.83%), 191.0201(21.89%)
H89	0.99	$C_4H_6O_5$	Malic acid	133.0143	133.0145	M-H	1.7	71.0140(33.41%), 72.9933(7.75%), 89.0246(5.67%), 115.0038(100.00%), 132.8683(13.90%), 133.0144(42.06%)

H90	1.23	$C_6H_8O_7$	Citric acid	191.0197	191.0199	M-H	1.03	87.0088(35.63%), 102.9489(13.38%), 111.0087(100.00%), 129.0193(7.14%), 146.9384(5.74%), 191.0197(22.95%)
H91	1.42	$C_4H_6O_4$	Methylmalonic acid	117.0193	117.0195	M-H	1.61	73.0295(80.09%), 74.0248(5.90%), 99.0088(7.76%), 99.9258(12.05%), 116.9285(100.00%), 117.0194(26.12%)
H92	1.54	$C_4H_8O_3$	3-Hydroxybutyric acid	103.0400	103.0402	M-H	1.69	57.0347(22.45%), 59.0139(100.00%), 73.0295(10.72%), 103.0401(59.44%)
H93	3.27	$C_7H_6O_4$	Protocatechuic acid	153.0193	153.0195	M-H	1.07	109.0295(100.00%), 153.0192(39.77%)
H94	4.17	$C_9H_8O_4$	Caffeic acid	163.0390	163.0391	M+H-H2O	0.80	89.0390(14.68%), 103.0545(23.01%), 107.0493(8.26%), 117.0336(24.35%), 131.0489(33.95%), 135.0439(69.64%), 145.0281(44.89%), 163.0386(100.00%)
H95	4.36	$C_6H_{10}O_3$	Ketoleucine	129.0557	129.0559	M-H	1.66	61.9884(58.03%), 85.0296(6.62%), 101.0608(5.75%), 129.0558(100.00%)
H96	4.71	$C_6H_{12}O_3$	Leucic acid	131.0714	131.0715	M-H	0.80	85.0659(81.10%), 131.0714(100.00%)
H97	5.00	$C_9H_{10}O_3$	3-(3-Hydroxyphenyl) propanoic acid	165.0557	165.0558	M-H	0.32	72.9932(13.53%), 119.0503(28.80%), 121.0659(19.14%), 147.0452(100.00%), 165.0556(51.63%)
H98	6.10	$C_{15}H_{20}O_4$	(S)-Abscisic acid	247.1329	247.1329	M+H-H2O	-0.02	201.1268(51.51%), 201.1633(100.00%), 205.1227(10.52%), 211.1119(5.34%), 229.1218(52.52%),

								247.1105(8.18%), 247.1336(54.10%)
H99	6.63	$C_8H_{16}O_3$	2-Hydroxyoctanoic acid	159.1027	159.1028	M-H	0.52	59.0140(22.82%), 69.0346(6.92%), 113.0973(90.36%), 158.0822(14.63%), 159.1026(100.00%)
**amino acids and derivatives**								
H100	0.77	$C_6H_{14}N_2O_2$	L-Lysine	147.1128	147.1128	M+H	0.08	84.0812(100.00%), 130.0862(35.68%)
H101	0.77	$C_5H_{12}N_2O_2$	Ornithine	133.0972	133.0973	M+H	0.73	70.0657(100.00%), 74.0242(7.77%), 115.0866(6.07%), 116.0706(42.06%)
H102	0.80	$C_9H_{20}N_2O_2$	N6,N6,N6-Trimethyl-L-lysine	189.1597	189.1598	M+H	0.40	84.0812(100.00%), 130.0861(99.74%), 143.1176(25.60%), 188.1387(47.14%), 189.1593(30.55%)
H103	0.81	$C_6H_9N_3O_2$	L-Histidine	154.0622	154.0624	M-H	1.25	96.9601(100.00%), 137.0356(13.09%), 154.0621(41.23%)
H104	0.94	$C_4H_8O_5$	L-Threonic acid	135.0299	135.0301	M-H	1.56	75.0088(100.00%), 89.0245(24.00%), 117.0193(9.42%), 134.8948(7.69%), 135.0300(80.73%)
H105	0.96	$C_4H_9N_3O_2$	Creatine	132.0767	132.0770	M+H	1.90	68.0501(5.43%), 71.0297(8.52%), 87.0444(6.00%), 91.0577(18.11%), 114.0662(100.00)
H106	0.96	$C_5H_9NO_2$	L-Proline	116.0706	116.0709	M+H	2.34	59.0499(6.18%), 70.0657(100.00%), 87.0445(8.66%),

								116.0706(26.77%)
H107	1.55	$C_6H_{13}NO_2$	L-Leucine	132.1019	132.1020	M+H	0.81	69.0704(5.20%), 86.0968(100.00%), 90.0553(10.35%), 132.0768(5.78%)
H108	2.40	$C_{15}H_{21}NO_7$	N-(1-Deoxy-1-fructosyl) phenylalanine	328.1391	328.1389	M+H	-0.54	120.0808(16.05%%), 132.0805(, 10.79%), 166.0865(13.75%), 264.1225(15.90%), 292.1176(29.62%), 310.1277(100.00)
H109	3.80	$C_{11}H_{12}N_2O_2$	L-Tryptophan	205.0971	205.0972	M+H	0.29	118.0653(6.42%), 144.0805(9.29%), 146.0598(56.47%), 159.0914(6.82%), 188.0703(100.00%)
H110	4.12	$C_{14}H_{18}N_2O_5$	Gamma-Glu-Phe	295.1288	295.1291	M+H	0.98	132.1019(11.22%), 166.0859(100.00%), 186.0910(6.65%), 232.0960(7.65%), 278.1014(10.19%), 295.1289(24.44%)
