# Supplementary material for: Unlocking the Anti-Breast Cancer Potential of Aralia chinensis L
Source: Curr Issues Mol Biol. 2025 Aug 16;47(8):662. doi: 10.3390/cimb47080662 (PMC12384973; doi:10.3390/cimb47080662)
Supplement: Supplementary file 1 [file cimb-47-00662-s001.zip › Table S3.pdf]

Table S3. Identification of absorbed prototype chemicals of TSAC in rat serum.

No.	Retention time	Formula	Identification	theoretical mass	Experimental mass	Adduct ion	Error (ppm)	Fragment Ions
M1	5.70	$C_{65}H_{104}O_{33}$	Elatoside L	1435.6352	1435.6348	M+Na	-0.29	439.3569(0.60%), 509.1472(30.83%), 965.5067(21.42%), 1435.6321(100.00%)
M2	6.04	$C_{47}H_{74}O_{19}$	Deslanoside	987.4808	987.4789	M+FA-H	-1.88	85.0296(12.25%), 119.0351(10.26%), 487.3445(1.15%), 941.4976(49.38%), 987.4807(100.00%)
M3	6.17	$C_{42}H_{66}O_{15}$	Elatoside H	809.4329	809.4325	M-H	-0.45	471.3478(0.29%), 587.3606(0.16%), 647.382(7.15%), 809.4324(100.00%)
M4	6.25	$C_{30}H_{48}O_{5}$	Caulophyllogenin	471.3469	471.3470	M+H-H_2O	0.24	391.3342(2.76%), 435.3241(9.96%), 453.3353(63.16%), 471.3458(100.00%)
M5	6.41	$C_{30}H_{46}O_{4}$	3*β*,21*α*-Dihydroxyoleana-11,13(18)-dien-29-oic acid	453.3364	453.3364	M+H-H_2O	0.06	389.3192(3.25%), 407.3303(7.88%), 435.3227(17.82%), 453.3338(100.00)
M6	6.74	$C_{41}H_{64}O_{14}$	Digoxin	825.4279	825.4270	M+FA-H	-1.03	101.0246(14.06%), 119.0350(6.14%), 779.4222(13.21%), 825.4262(100.00%)
M7	6.78	$C_{54}H_{88}O_{23}$	Congmunoside V	1149.5698	1149.5721	M+FA-H	2.04	455.3545(23.06%), 617.4058(14.03%),

								779.4579(11.14%), 941.5101(100.00%), 1103.5632(23.21%), 1149.5645(17.02%)
M8	6.78	$C_{48}H_{76}O_{19}$	Elatoside I	979.4873	979.4870	M+Na	-0.31	431.119(0.49%), 461.1211(0.17%), 523.2016(26.92), 641.4056(0.19%), 751.2085(0.07%), 761.5175(0.08%), 791.1583(0.07%), 803.4448(0.21%), 810.8768(0.07%), 819.6225(0.06%), 911.7361(0.06%), 914.277(0.07%), 979.4860(100.00%)
M9	6.85	$C_{54}H_{86}O_{24}$	Elatoside D	1141.5401	1141.5399	M+Na	-0.17	461.1254(0.02%), 509.1481(0.02%), 523.1258(50.37%), 541.1401(0.46%), 641.4012(1.61%), 969.4690(0.02%), 1141.5374(100.00%)
M10	7.01	$C_{30}H_{48}O_3$	Oleanolic acid	457.3676	457.3674	M+H	-0.46	393.3501(7.31%), 411.3612(100.00%), 439.3563(54.60%), 457.3666(41.68%)
M11	7.26	$C_{48}H_{76}O_{19}$	Spinasaponin A 28-O-glucoside	979.4873	979.4870	M+Na	-0.29	1121.1015(0.28%), 181.0119(0.22%), 379.0838(2.36%), 523.1293(1.32%),

								623.3983(0.14%), 641.4013(4.80%), 684.3849(0.14%), 803.4539(0.04%), 979.4858(100.00%)
M12	7.26	$C_{48}H_{76}O_{19}$	Sandosaponin A	955.4908	955.4900	M-H	-0.81	455.3532(3.25%), 569.3830(9.45%), 587.3931(1.51%), 613.3723(2.62%), 631.3846(1.29%), 731.4378(3.62%), 793.4350(10.30%), 955.4884(100.00%)
M13	7.28	$C_{48}H_{76}O_{19}$	Ginsenoside Ro	974.5319	974.5317	M+NH4	-0.18	191.1791(18.26%), 321.0806(14.02%), 339.0915(25.27%), 393.3505(5.43%), 439.3561(100.00%), 501.1448(2.43%), 795.4465(1.59%), 974.5319(2.18%)
M14	7.59	$C_{47}H_{74}O_{18}$	Araloside A	944.5213	944.5216	M+NH4	0.27	121.1011(2.23%), 201.1639(7.89%), 439.3562(100.00%), 471.1333(1.72%), 944.5149(3.49%)
M15	7.86	$C_{42}H_{66}O_{14}$	Chikusetsusaponin Ⅳa	793.4379	793.4375	M -H	-0.54	455.3531(0.19%), 569.3846(13.24%), 613.3764(2.06%), 631.3835(17.02%), 793.4346(100.00%)

M16	8.17	$C_{30}H_{48}O_4$	Echinocystic acid	455.3520	455.3519	M-H_2O-H	-0.25	391.3354(3.73%), 409.3452(3.39%), 437.3409(57.44%), 455.3510(100.00%)
M17	8.57	$C_{48}H_{78}O_{18}$	Congmuyanoside F	987.5171	987.5167	M+FA-H	-0.38	455.3528(100.00%), 647.3805(0.78%), 779.4538(13.28%), 941.4982(45.72%), 987.5134(33.39%)
M18	8.89	$C_{48}H_{78}O_{19}$	Congmuyenoside I	939.4959	939.4961	M-H_2O-H	0.22	455.3551(5.28%), 569.3849(16.15%), 575.5934(0.53%), 595.3659(4.63%), 648.5557(0.42%), 675.7114(0.42%), 694.1721(0.56%), 731.4381(13.59%), 775.4194(0.80%), 925.4836(0.77%), 939.4929(100.00%)
M19	9.12	$C_{47}H_{74}O_{18}$	Oleanolic acid-3-O-glucosyl(1-2) xylyl(1-3) gluco- siduronic acid	971.4857	971.4846	M+FA-H	-1.15	455.3506(2.05%), 569.3857(6.96%), 655.6432(0.75%), 731.4400(2.08%), 801.5801(0.70%), 925.4788(100.00%), 971.4691(0.82%)
M20	9.23	$C_{42}H_{66}O_{14}$	Zingibroside R1	812.4791	812.4784	M+NH_4	-0.82	184.0730(100.00%), 191.1788(11.33%), 339.0911(12.50%), 393.3514(3.03%),

								439.3564(55.18%), 484.5266(0.35%), 628.9764(0.40%), 812.4580(11.99%)
M21	9.91	$C_{41}H_{66}O_{14}$	Congmuyanoside A	763.4274	763.4270	M-H_2O-H	-0.51	455.3518(3.56%), 587.3929(0.13%), 631.3862(0.75%), 763.4263(100.00%)
M22	10.19	$C_{36}H_{56}O_9$	Calenduloside E	631.3851	631.3846	M-H	-0.81	455.3524(7.38%), 509.3621(1.97%), 511.3411(0.29%), 555.3693(2.34%), 571.3682(0.04%), 613.374(0.17%), 631.3838(100.00%)
