# Supplementary material for: Unlocking the Anti-Breast Cancer Potential of Aralia chinensis L
Source: Curr Issues Mol Biol. 2025 Aug 16;47(8):662. doi: 10.3390/cimb47080662 (PMC12384973; doi:10.3390/cimb47080662)
Supplement: Supplementary file 1 [file cimb-47-00662-s001.zip › Table S4.pdf]

Table S4. docking scores of 22 prototype components to 5 core targets

Compounds	6ATE_SRC	1H90_PIK3R1	2RDO_PIK3CA	6NJS_STAT3	1XKK_EGFR
3beta,21alpha-Dihydroxyoleana-11,13(18)-dien-29-oic acid	-3.5	-4.9	-4.5	-4.1	-4.2
Araloside A	-6.1	-7.3	-8.3	-7.9	-6.8
Calenduloside E	-4.6	-6.6	-6.7	-4.0	-6.1
Caulophyllogenin	-3.1	-5.0	-5.5	-4.4	-4.3
Chikusetsusaponin Ⅳa	-5.0	-6.5	-6.2	-5.1	-4.6
Congmunoside Ⅴ	-5.4	-6.6	-7.7	-6.5	-4.7
Congmuyanoside A	-5.2	-6.2	-7.8	-5.6	-4.6
Congmuyanoside F	-5.9	-6.7	-7.1	-6.3	-6.7
Congmuyenoside I	-6.4	-5.9	-5.3	-6.8	-5.1

Deslanoside	-6.1	-7.2	-7.2	-5.7	-6.0
Digoxin	-5.8	-5.7	-6.9	-3.0	-5.5
Echinocystic acid	-3.0	-4.7	-5.5	-6.2	-4.2
Elatoside D	-4.7	-6.7	-8.3	-5.1	-5.7
Elatoside H	-4.5	-8.4	-6.7	-4.2	-6.8
Elatoside I	-5.5	-6.8	-7.1	-6.1	-5.8
Elatoside L	-6.9	-7.3	-9.8	-4.9	-6.0
Ginsenoside Ro	-4.6	-8.0	-7.0	-3.3	-6.4
Oleanolic acid	-2.7	-4.4	-4.5	-4.3	-4.3
Oleanolic acid-3-O-glucosyl(1-2)xylyl(1-3)glucosiduronic acid	-3.9	-8.4	-6.9	-5.9	-6.2
Sandosaponin A	-4.6	-5.8	-7.0	-3.9	-5.4

SpinasaponinA 28-O-glucoside	-6.1	-6.2	-8.1	-7.0	-5.6
Zingibroside R1	-3.7	-7.7	-5.8	-4.6	-3.6
