# Supplementary figures and images for: Unlocking the Anti-Breast Cancer Potential of Aralia chinensis L
Source: Curr Issues Mol Biol. 2025 Aug 16;47(8):662. doi: 10.3390/cimb47080662 (PMC12384973; doi:10.3390/cimb47080662)

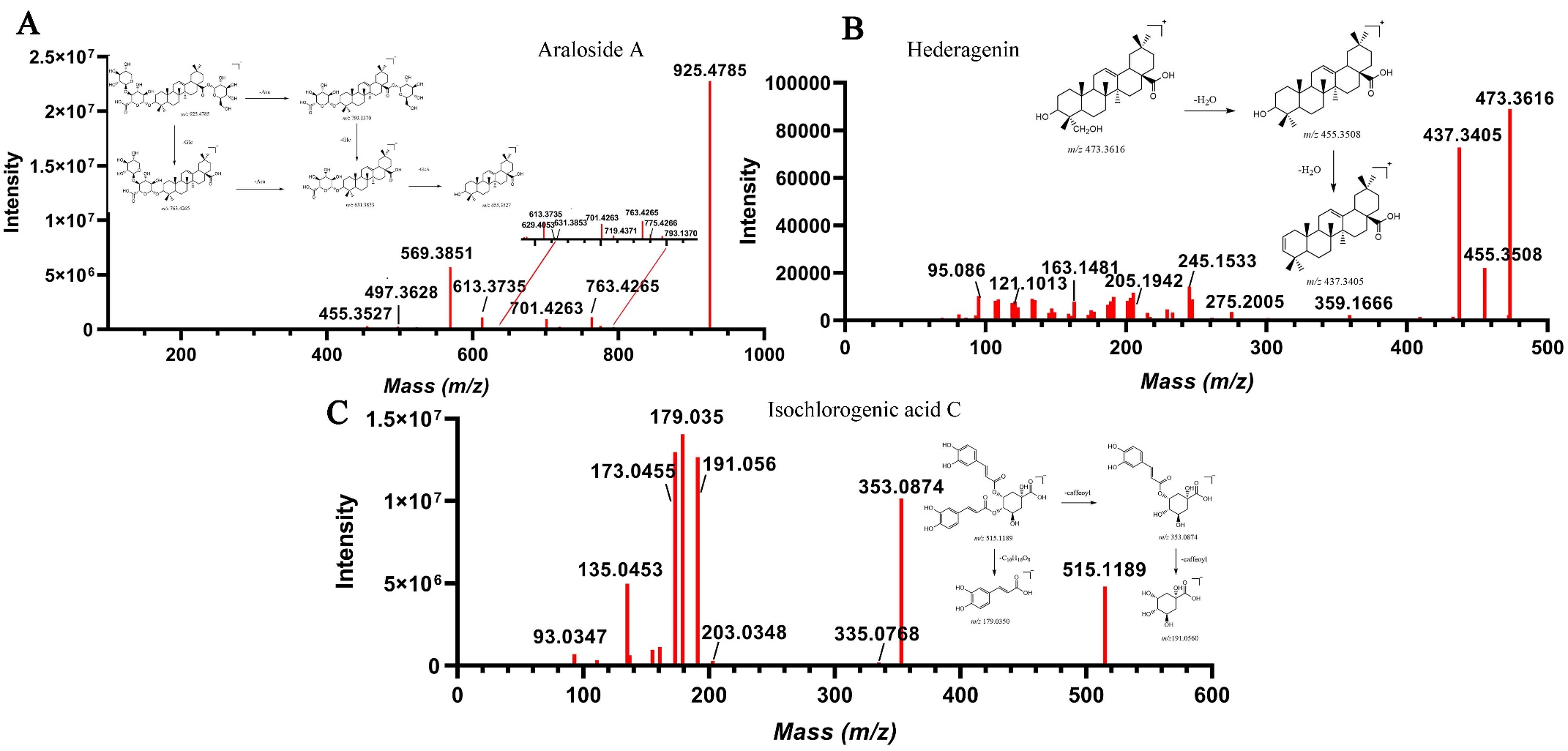

Supplement: Supplementary file 1 [file cimb-47-00662-s001.zip › Figure S1.tif]

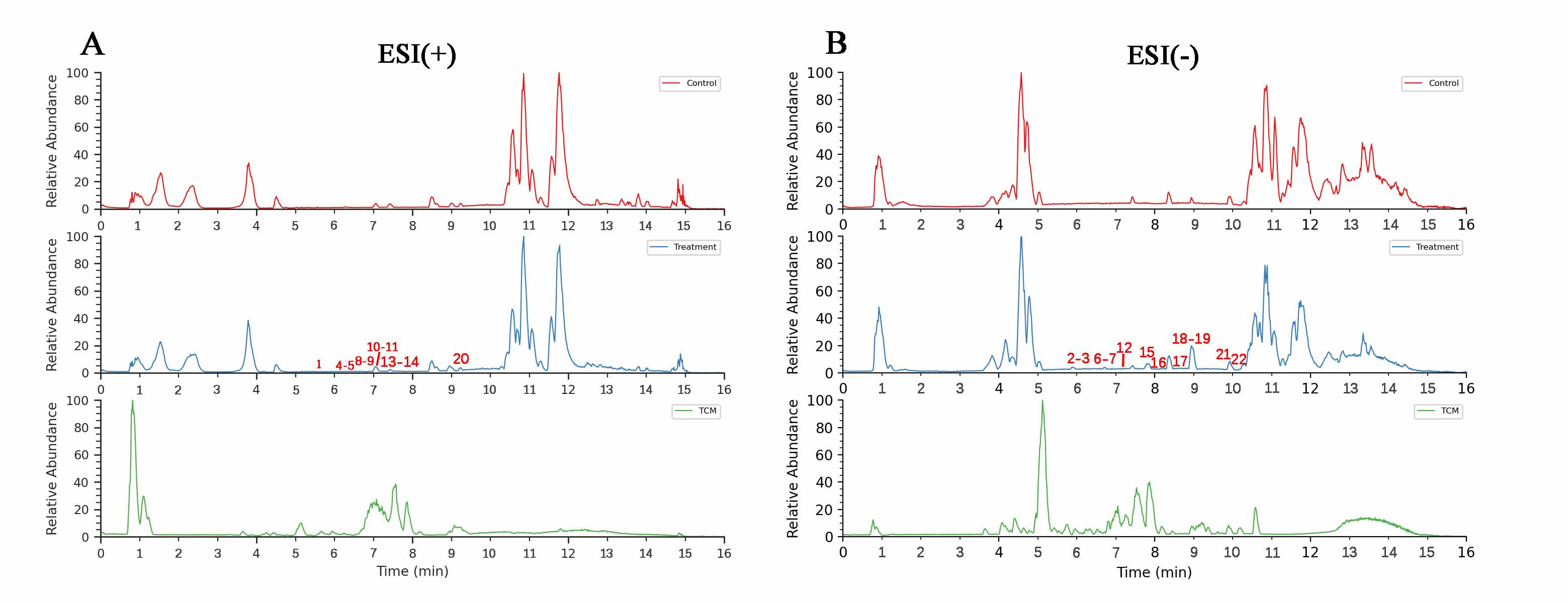

Supplement: Supplementary file 1 [file cimb-47-00662-s001.zip › Figure S2.tif]

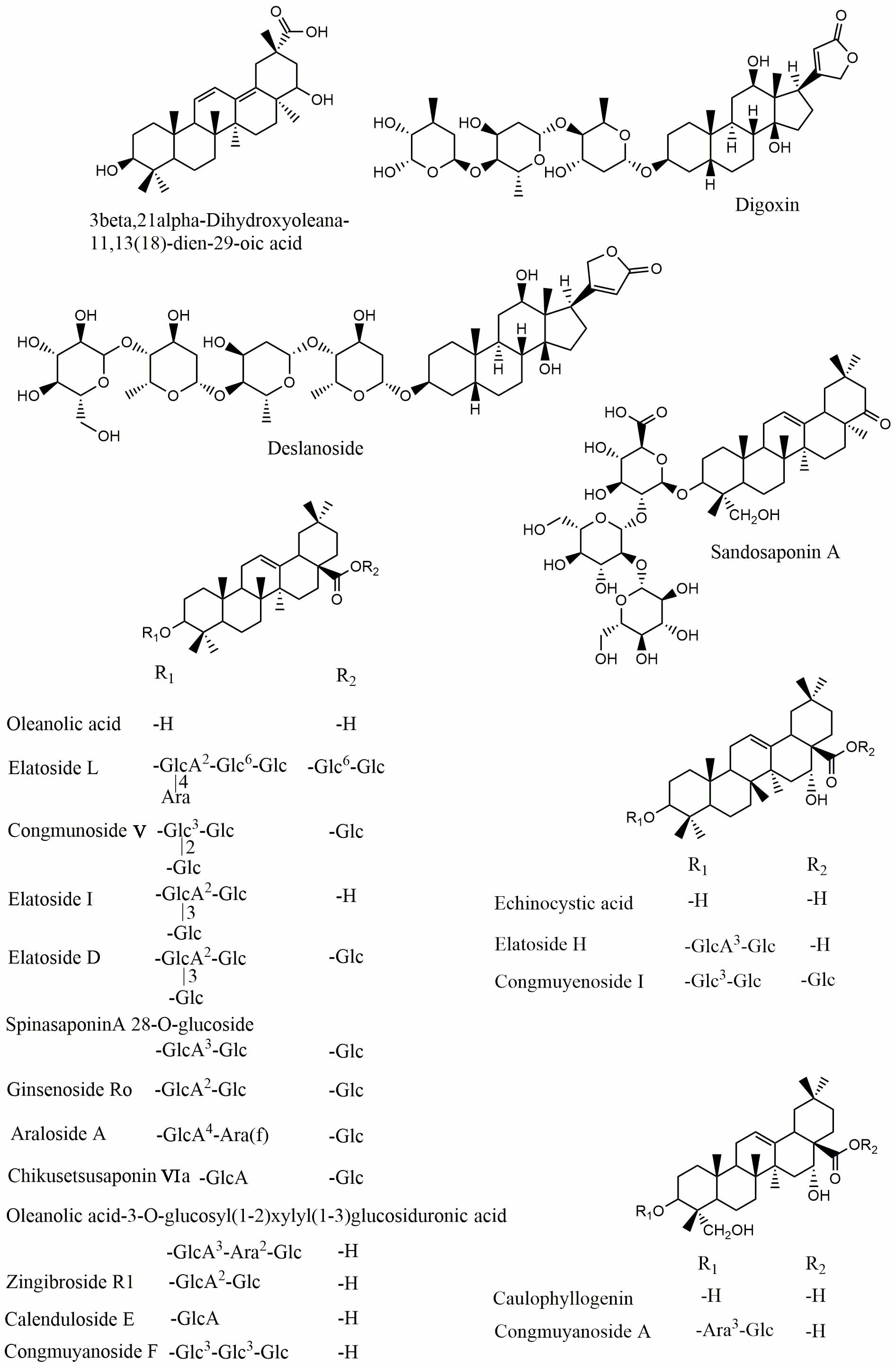

Supplement: Supplementary file 1 [file cimb-47-00662-s001.zip › Figure S3.tif]

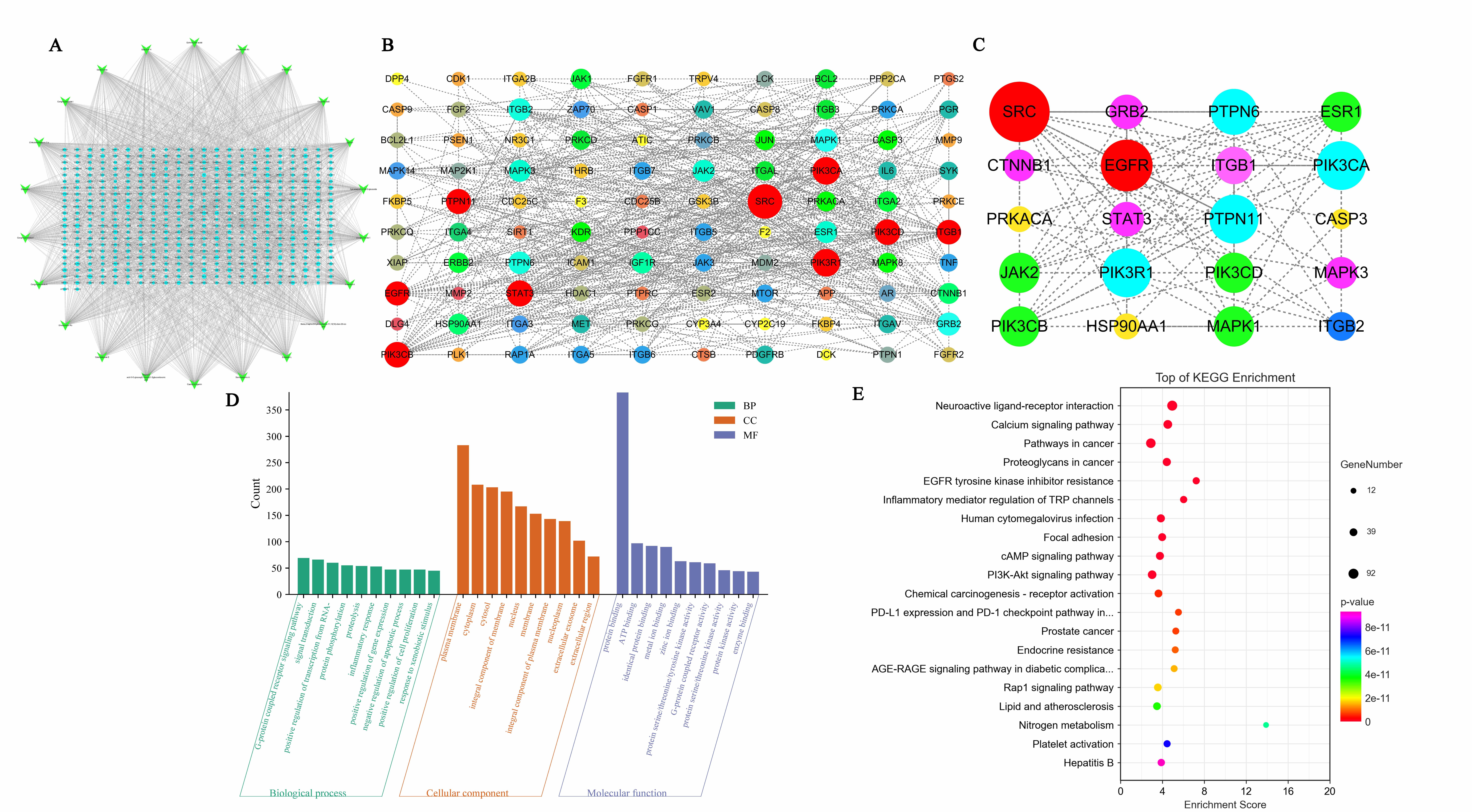

Supplement: Supplementary file 1 [file cimb-47-00662-s001.zip › Figure S4.tif]

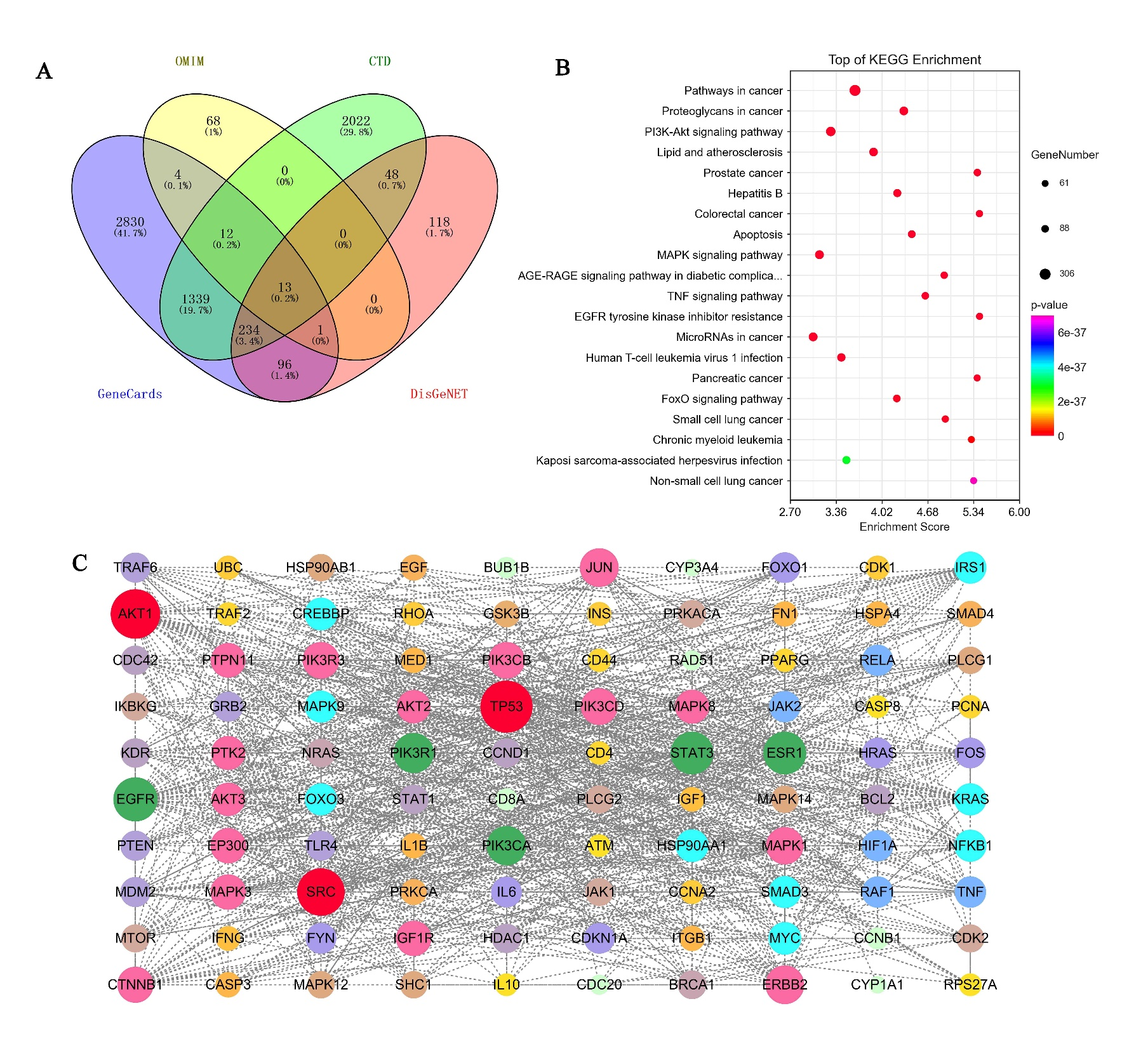

Supplement: Supplementary file 1 [file cimb-47-00662-s001.zip › Figure S5.tif]

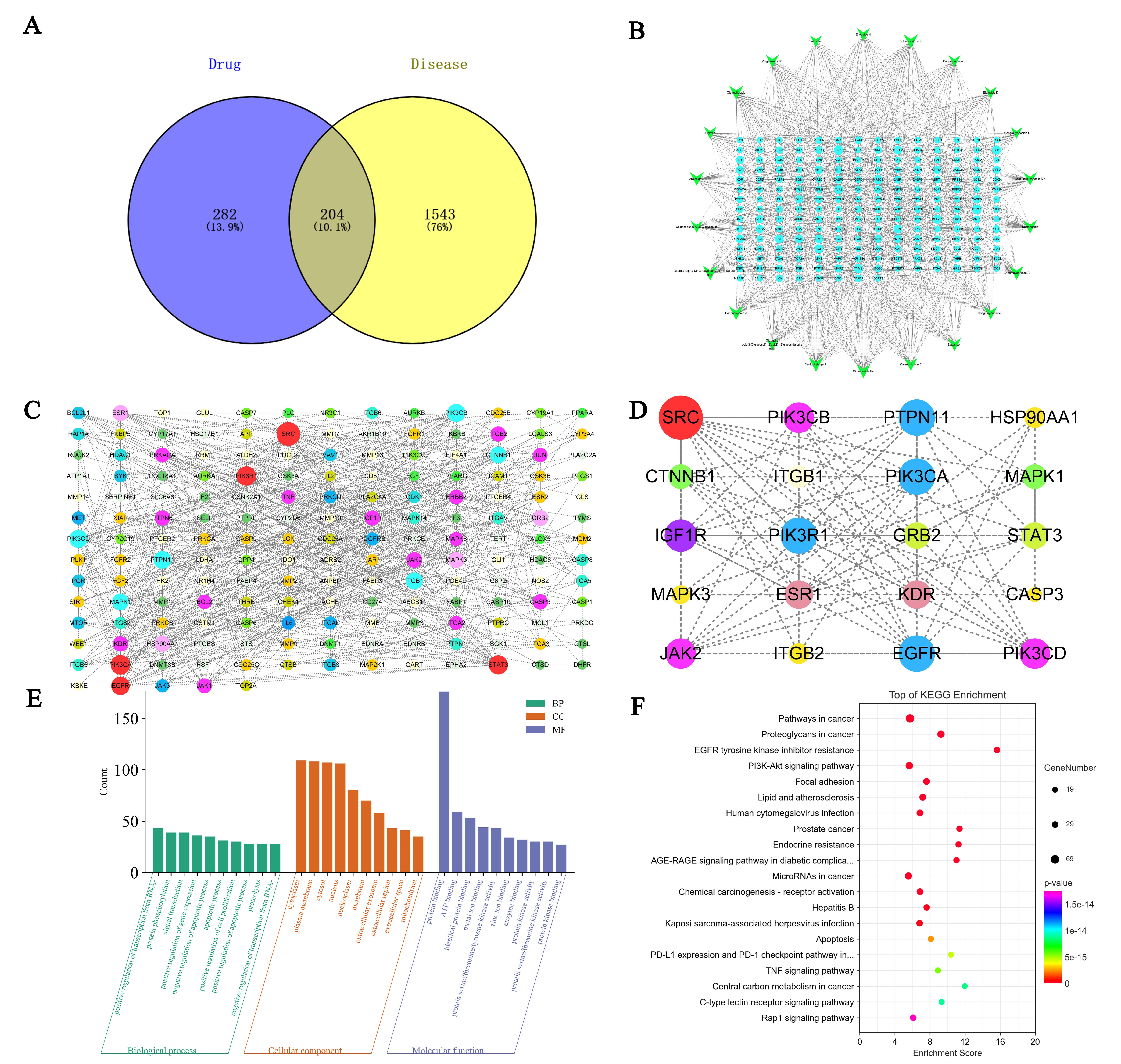

Supplement: Supplementary file 1 [file cimb-47-00662-s001.zip › Figure S6.tif]

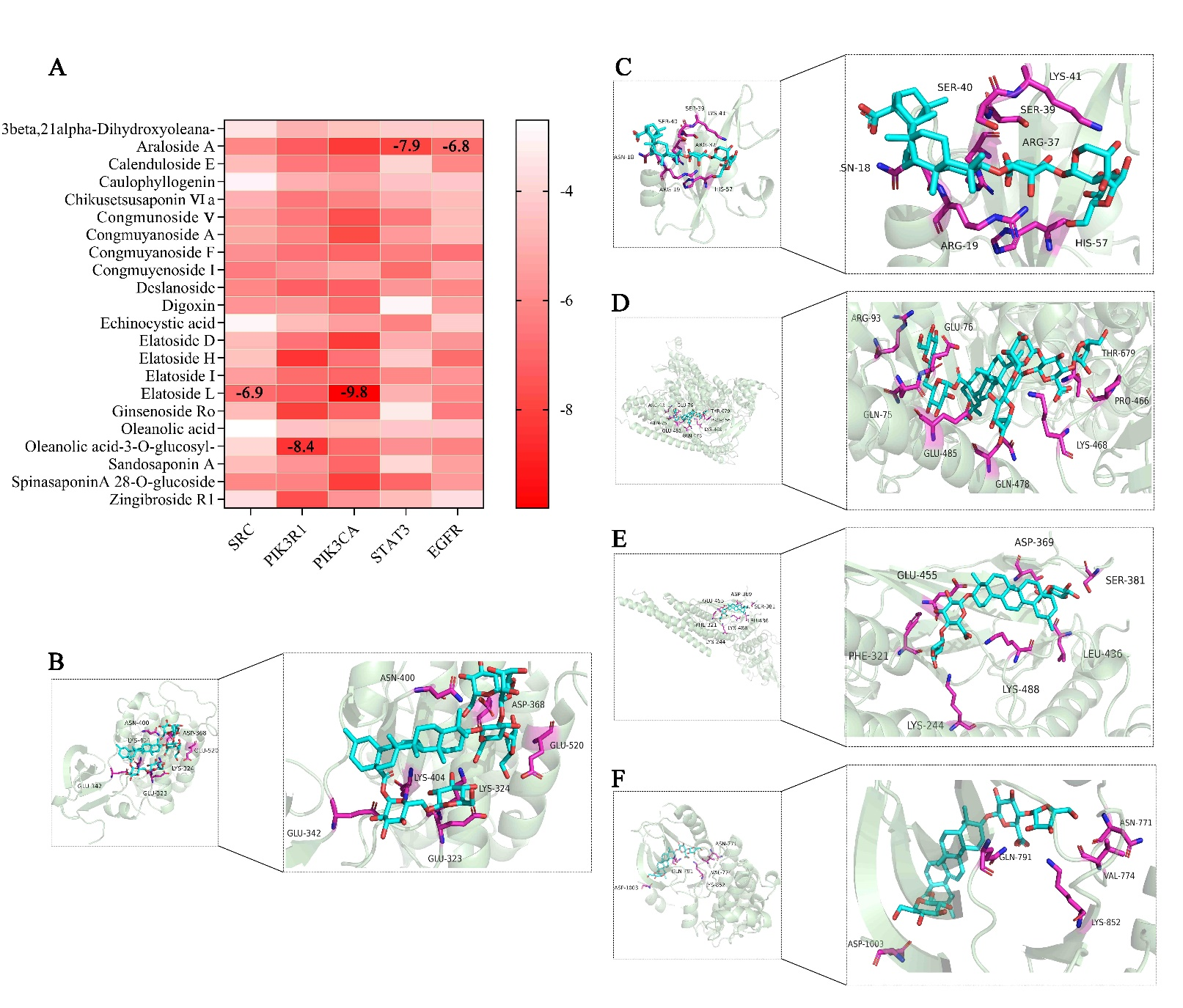

Supplement: Supplementary file 1 [file cimb-47-00662-s001.zip › Figure S7.tif]

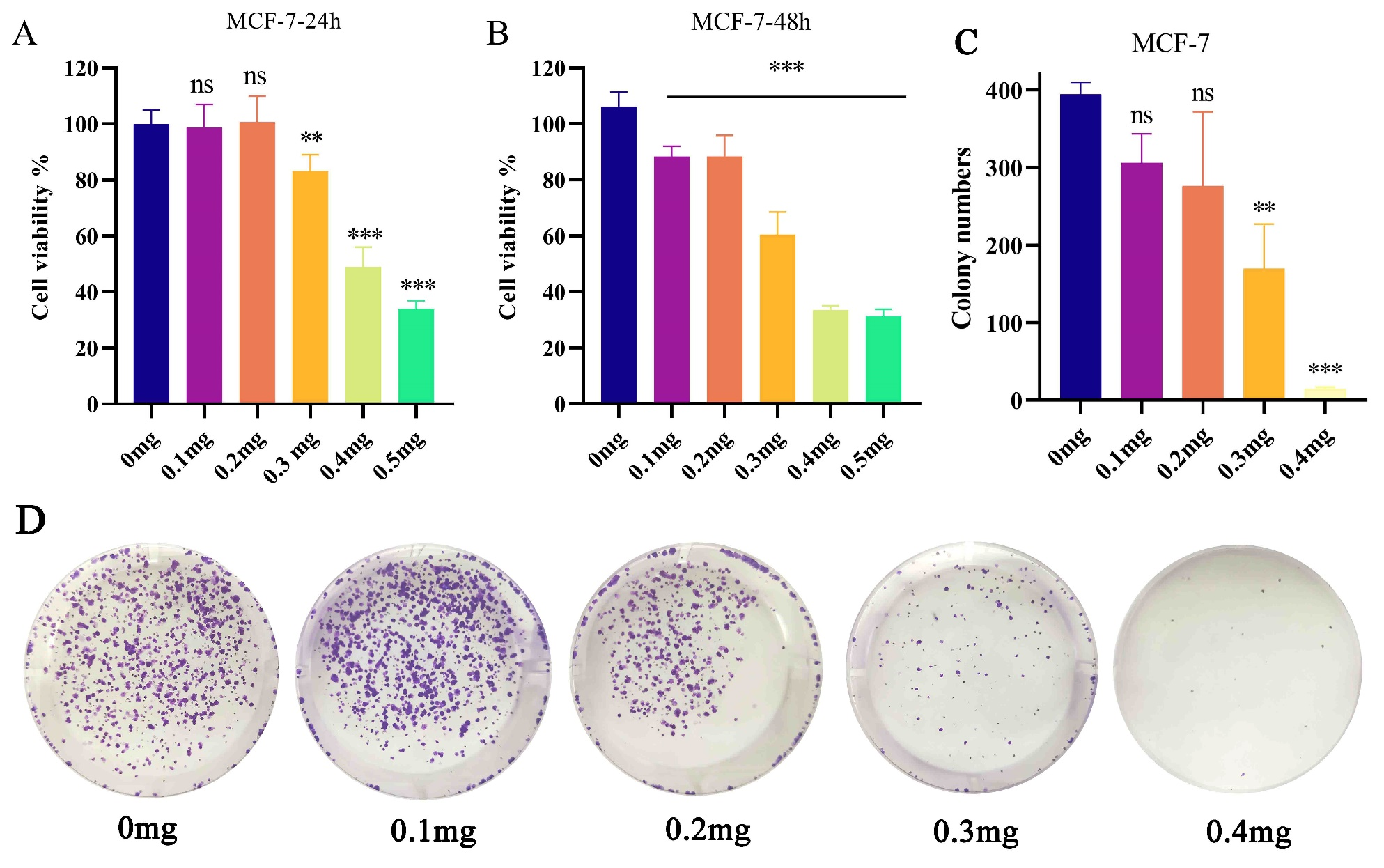

Supplement: Supplementary file 1 [file cimb-47-00662-s001.zip › Figure S8.tif]

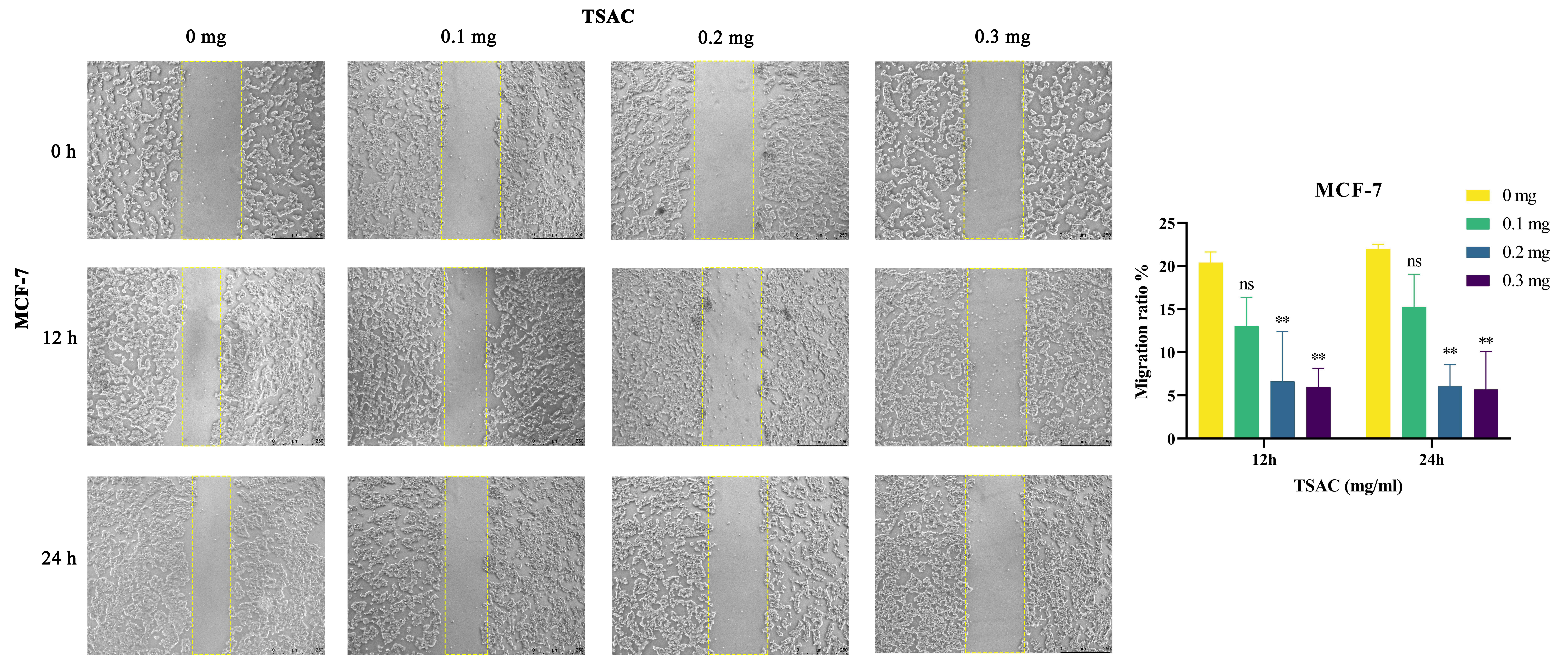

Supplement: Supplementary file 1 [file cimb-47-00662-s001.zip › Figure S9.tif]
